# Reweighting simulated events using machine-learning techniques in the CMS experiment

**DOI:** 10.1140/epjc/s10052-025-14097-x

**Published:** 2025-05-06

**Authors:** A. Hayrapetyan, A. Tumasyan, W. Adam, J. W. Andrejkovic, L. Benato, T. Bergauer, S. Chatterjee, K. Damanakis, M. Dragicevic, P. S. Hussain, M. Jeitler, N. Krammer, A. Li, D. Liko, I. Mikulec, J. Schieck, R. Schöfbeck, D. Schwarz, M. Sonawane, W. Waltenberger, C.-E. Wulz, T. Janssen, T. Van Laer, P. Van Mechelen, N. Breugelmans, J. D’Hondt, S. Dansana, A. De Moor, M. Delcourt, F. Heyen, S. Lowette, I. Makarenko, D. Müller, S. Tavernier, M. Tytgat, G. P. Van Onsem, S. Van Putte, D. Vannerom, B. Bilin, B. Clerbaux, A. K. Das, I. De Bruyn, G. De Lentdecker, H. Evard, L. Favart, P. Gianneios, J. Jaramillo, A. Khalilzadeh, F. A. Khan, K. Lee, A. Malara, S. Paredes, M. A. Shahzad, L. Thomas, M. Vanden Bemden, C. Vander Velde, P. Vanlaer, M. De Coen, D. Dobur, G. Gokbulut, Y. Hong, J. Knolle, L. Lambrecht, D. Marckx, K. Mota Amarilo, K. Skovpen, N. Van Den Bossche, J. van der Linden, L. Wezenbeek, A. Benecke, A. Bethani, G. Bruno, C. Caputo, J. De Favereau De Jeneret, C. Delaere, I. S. Donertas, A. Giammanco, A. O. Guzel, Sa. Jain, V. Lemaitre, J. Lidrych, P. Mastrapasqua, T. T. Tran, S. Turkcapar, G. A. Alves, E. Coelho, G. Correia Silva, C. Hensel, T. Menezes De Oliveira, C. Mora Herrera, P. Rebello Teles, M. Soeiro, E. J. Tonelli Manganote, A. Vilela Pereira, W. L. Aldá Júnior, M. Barroso Ferreira Filho, H. Brandao Malbouisson, W. Carvalho, J. Chinellato, E. M. Da Costa, G. G. Da Silveira, D. De Jesus Damiao, S. Fonseca De Souza, R. Gomes De Souza, T. Laux Kuhn, M. Macedo, J. Martins, L. Mundim, H. Nogima, J. P. Pinheiro, A. Santoro, A. Sznajder, M. Thiel, C. A. Bernardes, L. Calligaris, T. R. Fernandez Perez Tomei, E. M. Gregores, I. Maietto Silverio, P. G. Mercadante, S. F. Novaes, B. Orzari, Sandra S. Padula, A. Aleksandrov, G. Antchev, R. Hadjiiska, P. Iaydjiev, M. Misheva, M. Shopova, G. Sultanov, A. Dimitrov, L. Litov, B. Pavlov, P. Petkov, A. Petrov, E. Shumka, S. Keshri, D. Laroze, S. Thakur, T. Cheng, T. Javaid, L. Yuan, Z. Hu, Z. Liang, J. Liu, G. M. Chen, H. S. Chen, M. Chen, F. Iemmi, C. H. Jiang, A. Kapoor, H. Liao, Z.-A. Liu, R. Sharma, J. N. Song, J. Tao, C. Wang, J. Wang, Z. Wang, H. Zhang, J. Zhao, A. Agapitos, Y. Ban, S. Deng, B. Guo, C. Jiang, A. Levin, C. Li, Q. Li, Y. Mao, S. Qian, S. J. Qian, X. Qin, X. Sun, D. Wang, H. Yang, L. Zhang, Y. Zhao, C. Zhou, S. Yang, Z. You, K. Jaffel, N. Lu, G. Bauer, B. Li, K. Yi, J. Zhang, Y. Li, Z. Lin, C. Lu, M. Xiao, C. Avila, D. A. Barbosa Trujillo, A. Cabrera, C. Florez, J. Fraga, J. A. Reyes Vega, F. Ramirez, C. Rendón, M. Rodriguez, A. A. Ruales Barbosa, J. D. Ruiz Alvarez, D. Giljanovic, N. Godinovic, D. Lelas, A. Sculac, M. Kovac, A. Petkovic, T. Sculac, P. Bargassa, V. Brigljevic, B. K. Chitroda, D. Ferencek, K. Jakovcic, A. Starodumov, T. Susa, A. Attikis, K. Christoforou, A. Hadjiagapiou, C. Leonidou, J. Mousa, C. Nicolaou, L. Paizanos, F. Ptochos, P. A. Razis, H. Rykaczewski, H. Saka, A. Stepennov, M. Finger, M. Finger, A. Kveton, E. Ayala, E. Carrera Jarrin, B. El-mahdy, S. Khalil, E. Salama, M. Abdullah Al-Mashad, M. A. Mahmoud, K. Ehataht, M. Kadastik, T. Lange, S. Nandan, C. Nielsen, J. Pata, M. Raidal, L. Tani, C. Veelken, H. Kirschenmann, K. Osterberg, M. Voutilainen, S. Bharthuar, N. Bin Norjoharuddeen, E. Brücken, F. Garcia, P. Inkaew, K. T. S. Kallonen, T. Lampén, K. Lassila-Perini, S. Lehti, T. Lindén, M. Myllymäki, M. M. Rantanen, H. Siikonen, J. Tuominiemi, P. Luukka, H. Petrow, M. Besancon, F. Couderc, M. Dejardin, D. Denegri, J. L. Faure, F. Ferri, S. Ganjour, P. Gras, G. Hamel de Monchenault, M. Kumar, V. Lohezic, J. Malcles, F. Orlandi, L. Portales, A. Rosowsky, M. Ö. Sahin, A. Savoy-Navarro, P. Simkina, M. Titov, M. Tornago, F. Beaudette, G. Boldrini, P. Busson, A. Cappati, C. Charlot, M. Chiusi, T. D. Cuisset, F. Damas, O. Davignon, A. De Wit, I. T. Ehle, B. A. Fontana Santos Alves, S. Ghosh, A. Gilbert, R. Granier de Cassagnac, A. Hakimi, B. Harikrishnan, L. Kalipoliti, G. Liu, M. Nguyen, C. Ochando, R. Salerno, J. B. Sauvan, Y. Sirois, L. Urda Gómez, E. Vernazza, A. Zabi, A. Zghiche, J.-L. Agram, J. Andrea, D. Apparu, D. Bloch, J.-M. Brom, E. C. Chabert, C. Collard, S. Falke, U. Goerlach, R. Haeberle, A.-C. Le Bihan, M. Meena, O. Poncet, G. Saha, M. A. Sessini, P. Van Hove, P. Vaucelle, A. Di Florio, D. Amram, S. Beauceron, B. Blancon, G. Boudoul, N. Chanon, D. Contardo, P. Depasse, C. Dozen, H. El Mamouni, J. Fay, S. Gascon, M. Gouzevitch, C. Greenberg, G. Grenier, B. Ille, E. Jourd‘huy, I. B. Laktineh, M. Lethuillier, L. Mirabito, S. Perries, A. Purohit, M. Vander Donckt, P. Verdier, J. Xiao, A. Khvedelidze, I. Lomidze, Z. Tsamalaidze, V. Botta, S. Consuegra Rodríguez, L. Feld, K. Klein, M. Lipinski, D. Meuser, A. Pauls, D. Pérez Adán, N. Röwert, M. Teroerde, S. Diekmann, A. Dodonova, N. Eich, D. Eliseev, F. Engelke, J. Erdmann, M. Erdmann, P. Fackeldey, B. Fischer, T. Hebbeker, K. Hoepfner, F. Ivone, A. Jung, M. Y. Lee, F. Mausolf, M. Merschmeyer, A. Meyer, S. Mukherjee, D. Noll, F. Nowotny, A. Pozdnyakov, Y. Rath, W. Redjeb, F. Rehm, H. Reithler, V. Sarkisovi, A. Schmidt, C. Seth, A. Sharma, J. L. Spah, A. Stein, F. Torres Da Silva De Araujo, S. Wiedenbeck, S. Zaleski, C. Dziwok, G. Flügge, T. Kress, A. Nowack, O. Pooth, A. Stahl, T. Ziemons, A. Zotz, H. Aarup Petersen, M. Aldaya Martin, J. Alimena, S. Amoroso, Y. An, J. Bach, S. Baxter, M. Bayatmakou, H. Becerril Gonzalez, O. Behnke, A. Belvedere, F. Blekman, K. Borras, A. Campbell, A. Cardini, C. Cheng, F. Colombina, G. Eckerlin, D. Eckstein, L. I. Estevez Banos, E. Gallo, A. Geiser, V. Guglielmi, M. Guthoff, A. Hinzmann, L. Jeppe, B. Kaech, M. Kasemann, C. Kleinwort, R. Kogler, M. Komm, D. Krücker, W. Lange, D. Leyva Pernia, K. Lipka, W. Lohmann, F. Lorkowski, R. Mankel, I.-A. Melzer-Pellmann, M. Mendizabal Morentin, A. B. Meyer, G. Milella, K. Moral Figueroa, A. Mussgiller, L. P. Nair, J. Niedziela, A. Nürnberg, Y. Otarid, J. Park, E. Ranken, A. Raspereza, D. Rastorguev, J. Rübenach, L. Rygaard, A. Saggio, M. Scham, S. Schnake, P. Schütze, C. Schwanenberger, D. Selivanova, K. Sharko, M. Shchedrolosiev, D. Stafford, F. Vazzoler, A. Ventura Barroso, R. Walsh, D. Wang, Q. Wang, K. Wichmann, L. Wiens, C. Wissing, Y. Yang, A. Zimermmane Castro Santos, A. Albrecht, S. Albrecht, M. Antonello, S. Bein, S. Bollweg, M. Bonanomi, P. Connor, K. El Morabit, Y. Fischer, E. Garutti, A. Grohsjean, J. Haller, D. Hundhausen, H. R. Jabusch, G. Kasieczka, P. Keicher, R. Klanner, W. Korcari, T. Kramer, C. C. Kuo, V. Kutzner, F. Labe, J. Lange, A. Lobanov, C. Matthies, L. Moureaux, M. Mrowietz, A. Nigamova, Y. Nissan, A. Paasch, K. J. Pena Rodriguez, T. Quadfasel, B. Raciti, M. Rieger, D. Savoiu, J. Schindler, P. Schleper, M. Schröder, J. Schwandt, M. Sommerhalder, H. Stadie, G. Steinbrück, A. Tews, B. Wiederspan, M. Wolf, S. Brommer, E. Butz, T. Chwalek, A. Dierlamm, A. Droll, U. Elicabuk, N. Faltermann, M. Giffels, A. Gottmann, F. Hartmann, R. Hofsaess, M. Horzela, U. Husemann, J. Kieseler, M. Klute, R. Koppenhöfer, O. Lavoryk, J. M. Lawhorn, M. Link, A. Lintuluoto, S. Maier, S. Mitra, M. Mormile, Th. Müller, M. Neukum, M. Oh, E. Pfeffer, M. Presilla, G. Quast, K. Rabbertz, B. Regnery, N. Shadskiy, I. Shvetsov, H. J. Simonis, L. Sowa, L. Stockmeier, K. Tauqeer, M. Toms, N. Trevisani, R. F. Von Cube, M. Wassmer, S. Wieland, F. Wittig, R. Wolf, X. Zuo, G. Anagnostou, G. Daskalakis, A. Kyriakis, A. Papadopoulos, A. Stakia, P. Kontaxakis, G. Melachroinos, Z. Painesis, I. Papavergou, I. Paraskevas, N. Saoulidou, K. Theofilatos, E. Tziaferi, K. Vellidis, I. Zisopoulos, G. Bakas, T. Chatzistavrou, G. Karapostoli, K. Kousouris, I. Papakrivopoulos, E. Siamarkou, G. Tsipolitis, A. Zacharopoulou, I. Bestintzanos, I. Evangelou, C. Foudas, C. Kamtsikis, P. Katsoulis, P. Kokkas, P.G. Kosmoglou Kioseoglou, N. Manthos, I. Papadopoulos, J. Strologas, C. Hajdu, D. Horvath, K. Márton, A. J. Rádl, F. Sikler, V. Veszpremi, M. Csanád, K. Farkas, A. Fehérkuti, M. M. A. Gadallah, Á. Kadlecsik, P. Major, G. Pásztor, G. I. Veres, L. Olah, B. Ujvari, G. Bencze, S. Czellar, J. Molnar, Z. Szillasi, T. Csorgo, F. Nemes, T. Novak, S. Bansal, S. B. Beri, V. Bhatnagar, G. Chaudhary, S. Chauhan, N. Dhingra, A. Kaur, A. Kaur, H. Kaur, M. Kaur, S. Kumar, T. Sheokand, J. B. Singh, A. Singla, A. Ahmed, A. Bhardwaj, A. Chhetri, B. C. Choudhary, A. Kumar, A. Kumar, M. Naimuddin, K. Ranjan, M. K. Saini, S. Saumya, S. Baradia, S. Barman, S. Bhattacharya, S. Das Gupta, S. Dutta, S. Dutta, S. Sarkar, M. M. Ameen, P. K. Behera, S. C. Behera, S. Chatterjee, G. Dash, P. Jana, P. Kalbhor, S. Kamble, J. R. Komaragiri, D. Kumar, T. Mishra, B. Parida, P. R. Pujahari, N. R. Saha, A. Sharma, A. K. Sikdar, R. K. Singh, P. Verma, S. Verma, A. Vijay, S. Dugad, G. B. Mohanty, M. Shelake, P. Suryadevara, A. Bala, S. Banerjee, R. M. Chatterjee, M. Guchait, Sh. Jain, A. Jaiswal, S. Kumar, G. Majumder, K. Mazumdar, S. Parolia, A. Thachayath, S. Bahinipati, C. Kar, D. Maity, P. Mal, K. Naskar, A. Nayak, S. Nayak, K. Pal, P. Sadangi, S. K. Swain, S. Varghese, D. Vats, S. Acharya, A. Alpana, S. Dube, B. Gomber, P. Hazarika, B. Kansal, A. Laha, B. Sahu, S. Sharma, K. Y. Vaish, H. Bakhshiansohi, A. Jafari, M. Zeinali, S. Bashiri, S. Chenarani, S. M. Etesami, Y. Hosseini, M. Khakzad, E. Khazaie, M. Mohammadi Najafabadi, S. Tizchang, M. Felcini, M. Grunewald, M. Abbrescia, A. Colaleo, D. Creanza, B. D’Anzi, N. De Filippis, M. De Palma, W. Elmetenawee, N. Ferrara, L. Fiore, G. Iaselli, L. Longo, M. Louka, G. Maggi, M. Maggi, I. Margjeka, V. Mastrapasqua, S. My, S. Nuzzo, A. Pellecchia, A. Pompili, G. Pugliese, R. Radogna, D. Ramos, A. Ranieri, L. Silvestris, F. M. Simone, Ü. Sözbilir, A. Stamerra, D. Troiano, R. Venditti, P. Verwilligen, A. Zaza, G. Abbiendi, C. Battilana, D. Bonacorsi, P. Capiluppi, A. Castro, F. R. Cavallo, M. Cuffiani, G. M. Dallavalle, T. Diotalevi, F. Fabbri, A. Fanfani, D. Fasanella, P. Giacomelli, L. Giommi, C. Grandi, L. Guiducci, S. Lo Meo, M. Lorusso, L. Lunerti, S. Marcellini, G. Masetti, F. L. Navarria, G. Paggi, A. Perrotta, F. Primavera, A. M. Rossi, S. Rossi Tisbeni, T. Rovelli, G. P. Siroli, S. Costa, A. Di Mattia, A. Lapertosa, R. Potenza, A. Tricomi, C. Tuve, P. Assiouras, G. Barbagli, G. Bardelli, B. Camaiani, A. Cassese, R. Ceccarelli, V. Ciulli, C. Civinini, R. D’Alessandro, E. Focardi, T. Kello, G. Latino, P. Lenzi, M. Lizzo, M. Meschini, S. Paoletti, A. Papanastassiou, G. Sguazzoni, L. Viliani, L. Benussi, S. Bianco, S. Meola, D. Piccolo, M. Alves Gallo Pereira, F. Ferro, E. Robutti, S. Tosi, A. Benaglia, F. Brivio, F. Cetorelli, F. De Guio, M. E. Dinardo, P. Dini, S. Gennai, R. Gerosa, A. Ghezzi, P. Govoni, L. Guzzi, M. T. Lucchini, M. Malberti, S. Malvezzi, A. Massironi, D. Menasce, L. Moroni, M. Paganoni, S. Palluotto, D. Pedrini, A. Perego, B. S. Pinolini, G. Pizzati, S. Ragazzi, T. Tabarelli de Fatis, S. Buontempo, A. Cagnotta, F. Carnevali, N. Cavallo, F. Fabozzi, A. O. M. Iorio, L. Lista, P. Paolucci, B. Rossi, R. Ardino, P. Azzi, N. Bacchetta, D. Bisello, P. Bortignon, G. Bortolato, A. Bragagnolo, A. C. M. Bulla, R. Carlin, P. Checchia, T. Dorigo, F. Fanzago, U. Gasparini, S. Giorgetti, E. Lusiani, M. Margoni, A. T. Meneguzzo, M. Migliorini, J. Pazzini, P. Ronchese, R. Rossin, F. Simonetto, M. Tosi, A. Triossi, S. Ventura, M. Zanetti, P. Zotto, A. Zucchetta, G. Zumerle, A. Braghieri, S. Calzaferri, D. Fiorina, P. Montagna, V. Re, C. Riccardi, P. Salvini, I. Vai, P. Vitulo, S. Ajmal, M. E. Ascioti, G. M. Bilei, C. Carrivale, D. Ciangottini, L. Fanò, M. Magherini, V. Mariani, M. Menichelli, F. Moscatelli, A. Rossi, A. Santocchia, D. Spiga, T. Tedeschi, C. Aimè, C. A. Alexe, P. Asenov, P. Azzurri, G. Bagliesi, R. Bhattacharya, L. Bianchini, T. Boccali, E. Bossini, D. Bruschini, R. Castaldi, M. A. Ciocci, M. Cipriani, V. D’Amante, R. Dell’Orso, S. Donato, A. Giassi, F. Ligabue, A. C. Marini, D. Matos Figueiredo, A. Messineo, S. Mishra, V. K. Muraleedharan Nair Bindhu, M. Musich, F. Palla, A. Rizzi, G. Rolandi, S. Roy Chowdhury, T. Sarkar, A. Scribano, P. Spagnolo, R. Tenchini, G. Tonelli, N. Turini, F. Vaselli, A. Venturi, P. G. Verdini, C. Baldenegro Barrera, P. Barria, C. Basile, F. Cavallari, L. Cunqueiro Mendez, D. Del Re, E. Di Marco, M. Diemoz, F. Errico, R. Gargiulo, E. Longo, L. Martikainen, J. Mijuskovic, G. Organtini, F. Pandolfi, R. Paramatti, C. Quaranta, S. Rahatlou, C. Rovelli, F. Santanastasio, L. Soffi, V. Vladimirov, N. Amapane, R. Arcidiacono, S. Argiro, M. Arneodo, N. Bartosik, R. Bellan, A. Bellora, C. Biino, C. Borca, N. Cartiglia, M. Costa, R. Covarelli, N. Demaria, L. Finco, M. Grippo, B. Kiani, F. Legger, F. Luongo, C. Mariotti, L. Markovic, S. Maselli, A. Mecca, L. Menzio, P. Meridiani, E. Migliore, M. Monteno, R. Mulargia, M. M. Obertino, G. Ortona, L. Pacher, N. Pastrone, M. Pelliccioni, M. Ruspa, F. Siviero, V. Sola, A. Solano, A. Staiano, C. Tarricone, D. Trocino, G. Umoret, R. White, J. Babbar, S. Belforte, V. Candelise, M. Casarsa, F. Cossutti, K. De Leo, G. Della Ricca, S. Dogra, J. Hong, B. Kim, J. Kim, D. Lee, H. Lee, S. W. Lee, C. S. Moon, Y. D. Oh, M. S. Ryu, S. Sekmen, B. Tae, Y. C. Yang, M. S. Kim, G. Bak, P. Gwak, H. Kim, D. H. Moon, E. Asilar, J. Choi, D. Kim, T. J. Kim, J. A. Merlin, Y. Ryou, S. Choi, S. Han, B. Hong, K. Lee, K. S. Lee, S. Lee, J. Yoo, J. Goh, S. Yang, H. S. Kim, Y. Kim, S. Lee, J. Almond, J. H. Bhyun, J. Choi, J. Choi, W. Jun, J. Kim, Y. W. Kim, S. Ko, H. Kwon, H. Lee, J. Lee, J. Lee, B. H. Oh, S. B. Oh, H. Seo, U. K. Yang, I. Yoon, W. Jang, D. Y. Kang, Y. Kang, S. Kim, B. Ko, J. S. H. Lee, Y. Lee, I. C. Park, Y. Roh, I. J. Watson, S. Ha, K. Hwang, H. D. Yoo, M. Choi, M. R. Kim, H. Lee, Y. Lee, I. Yu, T. Beyrouthy, Y. Gharbia, F. Alazemi, K. Dreimanis, A. Gaile, C. Munoz Diaz, D. Osite, G. Pikurs, A. Potrebko, M. Seidel, D. Sidiropoulos Kontos, N. R. Strautnieks, M. Ambrozas, A. Juodagalvis, A. Rinkevicius, G. Tamulaitis, I. Yusuff, Z. Zolkapli, J. F. Benitez, A. Castaneda Hernandez, H. A. Encinas Acosta, L. G. Gallegos Maríñez, M. León Coello, J. A. Murillo Quijada, A. Sehrawat, L. Valencia Palomo, G. Ayala, H. Castilla-Valdez, H. Crotte Ledesma, E. De La Cruz-Burelo, I. Heredia-De La Cruz, R. Lopez-Fernandez, J. Mejia Guisao, C. A. Mondragon Herrera, A. Sánchez Hernández, C. Oropeza Barrera, D. L. Ramirez Guadarrama, M. Ramírez García, I. Bautista, I. Pedraza, H. A. Salazar Ibarguen, C. Uribe Estrada, I. Bubanja, N. Raicevic, P. H. Butler, A. Ahmad, M. I. Asghar, A. Awais, M. I. M. Awan, H. R. Hoorani, W. A. Khan, V. Avati, L. Grzanka, M. Malawski, H. Bialkowska, M. Bluj, M. Górski, M. Kazana, M. Szleper, P. Zalewski, K. Bunkowski, K. Doroba, A. Kalinowski, M. Konecki, J. Krolikowski, A. Muhammad, P. Fokow, K. Pozniak, W. Zabolotny, M. Araujo, D. Bastos, C. Beirão Da Cruz E Silva, A. Boletti, M. Bozzo, T. Camporesi, G. Da Molin, P. Faccioli, M. Gallinaro, J. Hollar, N. Leonardo, G. B. Marozzo, A. Petrilli, M. Pisano, J. Seixas, J. Varela, J. W. Wulff, P. Adzic, P. Milenovic, D. Devetak, M. Dordevic, J. Milosevic, L. Nadderd, V. Rekovic, J. Alcaraz Maestre, Cristina F. Bedoya, J. A. Brochero Cifuentes, Oliver M. Carretero, M. Cepeda, M. Cerrada, N. Colino, B. De La Cruz, A. Delgado Peris, A. Escalante Del Valle, D. Fernández Del Val, J. P. Fernández Ramos, J. Flix, M. C. Fouz, O. Gonzalez Lopez, S. Goy Lopez, J. M. Hernandez, M. I. Josa, J. Llorente Merino, C. Martin Perez, E. Martin Viscasillas, D. Moran, C. M. Morcillo Perez, Á. Navarro Tobar, C. Perez Dengra, A. Pérez-Calero Yzquierdo, J. Puerta Pelayo, I. Redondo, S. Sánchez Navas, J. Sastre, J. Vazquez Escobar, J. F. de Trocóniz, B. Alvarez Gonzalez, J. Cuevas, J. Fernandez Menendez, S. Folgueras, I. Gonzalez Caballero, P. Leguina, E. Palencia Cortezon, J. Prado Pico, C. Ramón Álvarez, V. Rodríguez Bouza, A. Soto Rodríguez, A. Trapote, C. Vico Villalba, P. Vischia, S. Bhowmik, S. Blanco Fernández, I. J. Cabrillo, A. Calderon, J. Duarte Campderros, M. Fernandez, G. Gomez, C. Lasaosa García, R. Lopez Ruiz, C. Martinez Rivero, P. Martinez Ruiz del Arbol, F. Matorras, P. Matorras Cuevas, E. Navarrete Ramos, J. Piedra Gomez, L. Scodellaro, I. Vila, J. M. Vizan Garcia, B. Kailasapathy, D. D. C. Wickramarathna, W. G. D. Dharmaratna, K. Liyanage, N. Perera, D. Abbaneo, C. Amendola, E. Auffray, G. Auzinger, J. Baechler, D. Barney, A. Bermúdez Martínez, M. Bianco, A. A. Bin Anuar, A. Bocci, L. Borgonovi, C. Botta, E. Brondolin, C. Caillol, G. Cerminara, N. Chernyavskaya, D. d’Enterria, A. Dabrowski, A. David, A. De Roeck, M. M. Defranchis, M. Deile, M. Dobson, G. Franzoni, W. Funk, S. Giani, D. Gigi, K. Gill, F. Glege, J. Hegeman, J. K. Heikkilä, B. Huber, V. Innocente, T. James, P. Janot, O. Kaluzinska, O. Karacheban, S. Laurila, P. Lecoq, E. Leutgeb, C. Lourenço, L. Malgeri, M. Mannelli, M. Matthewman, A. Mehta, F. Meijers, S. Mersi, E. Meschi, V. Milosevic, F. Monti, F. Moortgat, M. Mulders, I. Neutelings, S. Orfanelli, F. Pantaleo, G. Petrucciani, A. Pfeiffer, M. Pierini, H. Qu, D. Rabady, B. Ribeiro Lopes, F. Riti, M. Rovere, H. Sakulin, R. Salvatico, S. Sanchez Cruz, S. Scarfi, C. Schwick, M. Selvaggi, A. Sharma, K. Shchelina, P. Silva, P. Sphicas, A. G. Stahl Leiton, A. Steen, S. Summers, D. Treille, P. Tropea, D. Walter, J. Wanczyk, J. Wang, K. A. Wozniak, S. Wuchterl, P. Zehetner, P. Zejdl, W. D. Zeuner, T. Bevilacqua, L. Caminada, A. Ebrahimi, W. Erdmann, R. Horisberger, Q. Ingram, H. C. Kaestli, D. Kotlinski, C. Lange, M. Missiroli, L. Noehte, T. Rohe, A. Samalan, T. K. Aarrestad, M. Backhaus, G. Bonomelli, A. Calandri, C. Cazzaniga, K. Datta, P. De Bryas Dexmiers D‘archiac, A. De Cosa, G. Dissertori, M. Dittmar, M. Donegà, F. Eble, M. Galli, K. Gedia, F. Glessgen, C. Grab, N. Härringer, T. G. Harte, D. Hits, W. Lustermann, A.-M. Lyon, R. A. Manzoni, M. Marchegiani, L. Marchese, A. Mascellani, F. Nessi-Tedaldi, F. Pauss, V. Perovic, S. Pigazzini, B. Ristic, R. Seidita, J. Steggemann, A. Tarabini, D. Valsecchi, R. Wallny, C. Amsler, P. Bärtschi, M. F. Canelli, K. Cormier, M. Huwiler, W. Jin, A. Jofrehei, B. Kilminster, S. Leontsinis, S. P. Liechti, A. Macchiolo, P. Meiring, F. Meng, J. Motta, A. Reimers, P. Robmann, M. Senger, E. Shokr, F. Stäger, R. Tramontano, C. Adloff, D. Bhowmik, C. M. Kuo, W. Lin, P. K. Rout, P. C. Tiwari, L. Ceard, K. F. Chen, Z. G. Chen, A. De Iorio, W.-S. Hou, T. H. Hsu, Y. W. Kao, S. Karmakar, G. Kole, Y. Y. Li, R.-S. Lu, E. Paganis, X. F. Su, J. Thomas-Wilsker, L. S. Tsai, D. Tsionou, H. Y. Wu, E. Yazgan, C. Asawatangtrakuldee, N. Srimanobhas, V. Wachirapusitanand, D. Agyel, F. Boran, F. Dolek, I. Dumanoglu, E. Eskut, Y. Guler, E. Gurpinar Guler, C. Isik, O. Kara, A. Kayis Topaksu, U. Kiminsu, Y. Komurcu, G. Onengut, K. Ozdemir, A. Polatoz, B. Tali, U. G. Tok, E. Uslan, I. S. Zorbakir, G. Sokmen, M. Yalvac, B. Akgun, I. O. Atakisi, E. Gülmez, M. Kaya, O. Kaya, S. Tekten, A. Cakir, K. Cankocak, G. G. Dincer, S. Sen, O. Aydilek, B. Hacisahinoglu, I. Hos, B. Kaynak, S. Ozkorucuklu, O. Potok, H. Sert, C. Simsek, C. Zorbilmez, S. Cerci, B. Isildak, D. Sunar Cerci, T. Yetkin, A. Boyaryntsev, B. Grynyov, L. Levchuk, D. Anthony, J. J. Brooke, A. Bundock, F. Bury, E. Clement, D. Cussans, H. Flacher, M. Glowacki, J. Goldstein, H. F. Heath, M.-L. Holmberg, L. Kreczko, S. Paramesvaran, L. Robertshaw, V. J. Smith, K. Walkingshaw Pass, A. H. Ball, K. W. Bell, A. Belyaev, C. Brew, R. M. Brown, D. J. A. Cockerill, C. Cooke, A. Elliot, K. V. Ellis, K. Harder, S. Harper, J. Linacre, K. Manolopoulos, D. M. Newbold, E. Olaiya, D. Petyt, T. Reis, A. R. Sahasransu, G. Salvi, T. Schuh, C. H. Shepherd-Themistocleous, I. R. Tomalin, K. C. Whalen, T. Williams, I. Andreou, R. Bainbridge, P. Bloch, C. E. Brown, O. Buchmuller, C. A. Carrillo Montoya, G. S. Chahal, D. Colling, J. S. Dancu, I. Das, P. Dauncey, G. Davies, M. Della Negra, S. Fayer, G. Fedi, G. Hall, A. Howard, G. Iles, C. R. Knight, P. Krueper, J. Langford, K. H. Law, J. León Holgado, L. Lyons, A.-M. Magnan, B. Maier, S. Mallios, M. Mieskolainen, J. Nash, M. Pesaresi, P. B. Pradeep, B. C. Radburn-Smith, A. Richards, A. Rose, K. Savva, C. Seez, R. Shukla, A. Tapper, K. Uchida, G. P. Uttley, T. Virdee, M. Vojinovic, N. Wardle, D. Winterbottom, J. E. Cole, A. Khan, P. Kyberd, I. D. Reid, S. Abdullin, A. Brinkerhoff, E. Collins, M. R. Darwish, J. Dittmann, K. Hatakeyama, V. Hegde, J. Hiltbrand, B. McMaster, J. Samudio, S. Sawant, C. Sutantawibul, J. Wilson, R. Bartek, A. Dominguez, A. E. Simsek, S. S. Yu, B. Bam, A. Buchot Perraguin, R. Chudasama, S. I. Cooper, C. Crovella, S. V. Gleyzer, E. Pearson, C. U. Perez, P. Rumerio, E. Usai, R. Yi, A. Akpinar, C. Cosby, G. De Castro, Z. Demiragli, C. Erice, C. Fangmeier, C. Fernandez Madrazo, E. Fontanesi, D. Gastler, F. Golf, S. Jeon, J. O‘cain, I. Reed, J. Rohlf, K. Salyer, D. Sperka, D. Spitzbart, I. Suarez, A. Tsatsos, A. G. Zecchinelli, G. Barone, G. Benelli, D. Cutts, L. Gouskos, M. Hadley, U. Heintz, K. W. Ho, J. M. Hogan, T. Kwon, G. Landsberg, K. T. Lau, J. Luo, S. Mondal, T. Russell, S. Sagir, X. Shen, F. Simpson, M. Stamenkovic, N. Venkatasubramanian, S. Abbott, B. Barton, C. Brainerd, R. Breedon, H. Cai, M. Calderon De La Barca Sanchez, M. Chertok, M. Citron, J. Conway, P. T. Cox, R. Erbacher, F. Jensen, O. Kukral, G. Mocellin, M. Mulhearn, S. Ostrom, W. Wei, S. Yoo, F. Zhang, K. Adamidis, M. Bachtis, D. Campos, R. Cousins, A. Datta, G. Flores Avila, J. Hauser, M. Ignatenko, M. A. Iqbal, T. Lam, Y. F. Lo, E. Manca, A. Nunez Del Prado, D. Saltzberg, V. Valuev, R. Clare, J. W. Gary, G. Hanson, A. Aportela, A. Arora, J. G. Branson, S. Cittolin, S. Cooperstein, D. Diaz, J. Duarte, L. Giannini, Y. Gu, J. Guiang, R. Kansal, V. Krutelyov, R. Lee, J. Letts, M. Masciovecchio, F. Mokhtar, S. Mukherjee, M. Pieri, D. Primosch, M. Quinnan, B. V. Sathia Narayanan, V. Sharma, M. Tadel, E. Vourliotis, F. Würthwein, Y. Xiang, A. Yagil, A. Barzdukas, L. Brennan, C. Campagnari, K. Downham, C. Grieco, M. M. Hussain, J. Incandela, J. Kim, A. J. Li, P. Masterson, H. Mei, J. Richman, S. N. Santpur, U. Sarica, R. Schmitz, F. Setti, J. Sheplock, D. Stuart, T. Á. Vámi, S. Wang, X. Yan, D. Zhang, S. Bhattacharya, A. Bornheim, O. Cerri, A. Latorre, J. Mao, H. B. Newman, G. Reales Gutiérrez, M. Spiropulu, J. R. Vlimant, C. Wang, S. Xie, R. Y. Zhu, J. Alison, S. An, P. Bryant, M. Cremonesi, V. Dutta, T. Ferguson, T. A. Gómez Espinosa, A. Harilal, A. Kallil Tharayil, C. Liu, T. Mudholkar, S. Murthy, P. Palit, K. Park, M. Paulini, A. Roberts, A. Sanchez, W. Terrill, J. P. Cumalat, W. T. Ford, A. Hart, A. Hassani, G. Karathanasis, N. Manganelli, J. Pearkes, C. Savard, N. Schonbeck, K. Stenson, K. A. Ulmer, S. R. Wagner, N. Zipper, D. Zuolo, J. Alexander, S. Bright-Thonney, X. Chen, D. J. Cranshaw, J. Dickinson, J. Fan, X. Fan, S. Hogan, P. Kotamnives, J. Monroy, M. Oshiro, J. R. Patterson, M. Reid, A. Ryd, J. Thom, P. Wittich, R. Zou, M. Albrow, M. Alyari, O. Amram, G. Apollinari, A. Apresyan, L. A. T. Bauerdick, D. Berry, J. Berryhill, P. C. Bhat, K. Burkett, J. N. Butler, A. Canepa, G. B. Cerati, H. W. K. Cheung, F. Chlebana, G. Cummings, I. Dutta, V. D. Elvira, Y. Feng, J. Freeman, A. Gandrakota, Z. Gecse, L. Gray, D. Green, A. Grummer, S. Grünendahl, D. Guerrero, O. Gutsche, R. M. Harris, R. Heller, T. C. Herwig, J. Hirschauer, B. Jayatilaka, S. Jindariani, M. Johnson, U. Joshi, T. Klijnsma, B. Klima, K. H. M. Kwok, S. Lammel, C. Lee, D. Lincoln, R. Lipton, T. Liu, C. Madrid, K. Maeshima, C. Mantilla, D. Mason, P. McBride, P. Merkel, S. Mrenna, S. Nahn, J. Ngadiuba, D. Noonan, S. Norberg, V. Papadimitriou, N. Pastika, K. Pedro, C. Pena, F. Ravera, A. Reinsvold Hall, L. Ristori, M. Safdari, E. Sexton-Kennedy, N. Smith, A. Soha, L. Spiegel, S. Stoynev, J. Strait, L. Taylor, S. Tkaczyk, N. V. Tran, L. Uplegger, E. W. Vaandering, I. Zoi, C. Aruta, P. Avery, D. Bourilkov, P. Chang, V. Cherepanov, R. D. Field, C. Huh, E. Koenig, M. Kolosova, J. Konigsberg, A. Korytov, K. Matchev, N. Menendez, G. Mitselmakher, K. Mohrman, A. Muthirakalayil Madhu, N. Rawal, S. Rosenzweig, Y. Takahashi, J. Wang, T. Adams, A. Al Kadhim, A. Askew, S. Bower, R. Hashmi, R. S. Kim, S. Kim, T. Kolberg, G. Martinez, H. Prosper, P. R. Prova, M. Wulansatiti, R. Yohay, J. Zhang, B. Alsufyani, S. Butalla, S. Das, T. Elkafrawy, M. Hohlmann, E. Yanes, M. R. Adams, A. Baty, C. Bennett, R. Cavanaugh, R. Escobar Franco, O. Evdokimov, C. E. Gerber, M. Hawksworth, A. Hingrajiya, D. J. Hofman, J. H. Lee, D. S. Lemos, A. H. Merrit, C. Mills, S. Nanda, G. Oh, B. Ozek, D. Pilipovic, R. Pradhan, E. Prifti, T. Roy, S. Rudrabhatla, N. Singh, M. B. Tonjes, N. Varelas, M. A. Wadud, Z. Ye, J. Yoo, M. Alhusseini, D. Blend, K. Dilsiz, L. Emediato, G. Karaman, O. K. Köseyan, J.-P. Merlo, A. Mestvirishvili, O. Neogi, H. Ogul, Y. Onel, A. Penzo, C. Snyder, E. Tiras, B. Blumenfeld, L. Corcodilos, J. Davis, A. V. Gritsan, L. Kang, S. Kyriacou, P. Maksimovic, M. Roguljic, J. Roskes, S. Sekhar, M. Swartz, A. Abreu, L. F. Alcerro Alcerro, J. Anguiano, S. Arteaga Escatel, P. Baringer, A. Bean, Z. Flowers, D. Grove, J. King, G. Krintiras, M. Lazarovits, C. Le Mahieu, J. Marquez, M. Murray, M. Nickel, M. Pitt, S. Popescu, C. Rogan, C. Royon, S. Sanders, C. Smith, G. Wilson, B. Allmond, R. Gujju Gurunadha, A. Ivanov, K. Kaadze, Y. Maravin, J. Natoli, D. Roy, G. Sorrentino, A. Baden, A. Belloni, J. Bistany-riebman, Y. M. Chen, S. C. Eno, N. J. Hadley, S. Jabeen, R. G. Kellogg, T. Koeth, B. Kronheim, Y. Lai, S. Lascio, A. C. Mignerey, S. Nabili, C. Palmer, C. Papageorgakis, M. M. Paranjpe, E. Popova, A. Shevelev, L. Wang, J. Bendavid, I. A. Cali, P. C. Chou, M. D’Alfonso, J. Eysermans, C. Freer, G. Gomez-Ceballos, M. Goncharov, G. Grosso, P. Harris, D. Hoang, D. Kovalskyi, J. Krupa, L. Lavezzo, Y.-J. Lee, K. Long, C. Mcginn, A. Novak, M. I. Park, C. Paus, C. Reissel, C. Roland, G. Roland, S. Rothman, G. S. F. Stephans, Z. Wang, B. Wyslouch, T. J. Yang, B. Crossman, B. M. Joshi, C. Kapsiak, M. Krohn, D. Mahon, J. Mans, B. Marzocchi, M. Revering, R. Rusack, R. Saradhy, N. Strobbe, K. Bloom, D. R. Claes, G. Haza, J. Hossain, C. Joo, I. Kravchenko, A. Rohilla, J. E. Siado, W. Tabb, A. Vagnerini, A. Wightman, F. Yan, D. Yu, H. Bandyopadhyay, L. Hay, H. W. Hsia, I. Iashvili, A. Kalogeropoulos, A. Kharchilava, M. Morris, D. Nguyen, S. Rappoccio, H. Rejeb Sfar, A. Williams, P. Young, G. Alverson, E. Barberis, J. Bonilla, B. Bylsma, M. Campana, J. Dervan, Y. Haddad, Y. Han, I. Israr, A. Krishna, J. Li, M. Lu, G. Madigan, R. Mccarthy, D. M. Morse, V. Nguyen, T. Orimoto, A. Parker, L. Skinnari, E. Tsai, D. Wood, J. Bueghly, S. Dittmer, K. A. Hahn, D. Li, Y. Liu, M. Mcginnis, Y. Miao, D. G. Monk, M. H. Schmitt, A. Taliercio, M. Velasco, G. Agarwal, R. Band, R. Bucci, S. Castells, A. Das, R. Goldouzian, M. Hildreth, K. Hurtado Anampa, T. Ivanov, C. Jessop, K. Lannon, J. Lawrence, N. Loukas, L. Lutton, J. Mariano, N. Marinelli, I. Mcalister, T. McCauley, C. Mcgrady, C. Moore, Y. Musienko, H. Nelson, M. Osherson, A. Piccinelli, R. Ruchti, A. Townsend, Y. Wan, M. Wayne, H. Yockey, M. Zarucki, L. Zygala, A. Basnet, M. Carrigan, L. S. Durkin, C. Hill, M. Joyce, M. Nunez Ornelas, K. Wei, D. A. Wenzl, B. L. Winer, B. R. Yates, H. Bouchamaoui, K. Coldham, P. Das, G. Dezoort, P. Elmer, A. Frankenthal, B. Greenberg, N. Haubrich, K. Kennedy, G. Kopp, S. Kwan, D. Lange, A. Loeliger, D. Marlow, I. Ojalvo, J. Olsen, D. Stickland, C. Tully, L. H. Vage, S. Malik, R. Sharma, A. S. Bakshi, S. Chandra, R. Chawla, A. Gu, L. Gutay, M. Jones, A. W. Jung, A. M. Koshy, M. Liu, G. Negro, N. Neumeister, G. Paspalaki, S. Piperov, V. Scheurer, J. F. Schulte, M. Stojanovic, J. Thieman, A. K. Virdi, F. Wang, A. Wildridge, W. Xie, Y. Yao, J. Dolen, N. Parashar, A. Pathak, D. Acosta, T. Carnahan, K. M. Ecklund, P. J. Fernández Manteca, S. Freed, P. Gardner, F. J. M. Geurts, I. Krommydas, W. Li, J. Lin, O. Miguel Colin, B. P. Padley, R. Redjimi, J. Rotter, E. Yigitbasi, Y. Zhang, A. Bodek, P. de Barbaro, R. Demina, J. L. Dulemba, A. Garcia-Bellido, O. Hindrichs, A. Khukhunaishvili, N. Parmar, P. Parygin, R. Taus, B. Chiarito, J. P. Chou, S. V. Clark, D. Gadkari, Y. Gershtein, E. Halkiadakis, M. Heindl, C. Houghton, D. Jaroslawski, S. Konstantinou, I. Laflotte, A. Lath, R. Montalvo, K. Nash, J. Reichert, H. Routray, P. Saha, S. Salur, S. Schnetzer, S. Somalwar, R. Stone, S. A. Thayil, S. Thomas, J. Vora, H. Wang, D. Ally, A. G. Delannoy, S. Fiorendi, S. Higginbotham, T. Holmes, A. R. Kanuganti, N. Karunarathna, L. Lee, E. Nibigira, S. Spanier, D. Aebi, M. Ahmad, T. Akhter, K. Androsov, O. Bouhali, R. Eusebi, J. Gilmore, T. Huang, T. Kamon, H. Kim, S. Luo, R. Mueller, D. Overton, D. Rathjens, A. Safonov, N. Akchurin, J. Damgov, N. Gogate, A. Hussain, Y. Kazhykarim, K. Lamichhane, S. W. Lee, A. Mankel, T. Peltola, I. Volobouev, E. Appelt, Y. Chen, S. Greene, A. Gurrola, W. Johns, R. Kunnawalkam Elayavalli, A. Melo, F. Romeo, P. Sheldon, S. Tuo, J. Velkovska, J. Viinikainen, B. Cardwell, H. Chung, B. Cox, J. Hakala, R. Hirosky, A. Ledovskoy, C. Neu, S. Bhattacharya, P. E. Karchin, A. Aravind, S. Banerjee, K. Black, T. Bose, E. Chavez, S. Dasu, P. Everaerts, C. Galloni, H. He, M. Herndon, A. Herve, C. K. Koraka, A. Lanaro, R. Loveless, J. Madhusudanan Sreekala, A. Mallampalli, A. Mohammadi, S. Mondal, G. Parida, L. Pétré, D. Pinna, A. Savin, V. Shang, V. Sharma, W. H. Smith, D. Teague, H. F. Tsoi, W. Vetens, A. Warden, S. Afanasiev, V. Alexakhin, D. Budkouski, I. Golutvin, I. Gorbunov, V. Karjavine, V. Korenkov, A. Lanev, A. Malakhov, V. Matveev, V. Palichik, V. Perelygin, M. Savina, V. Shalaev, S. Shmatov, S. Shulha, V. Smirnov, O. Teryaev, N. Voytishin, B. S. Yuldashev, A. Zarubin, I. Zhizhin, Yu. Andreev, A. Dermenev, S. Gninenko, N. Golubev, A. Karneyeu, D. Kirpichnikov, M. Kirsanov, N. Krasnikov, I. Tlisova, A. Toropin, G. Gavrilov, V. Golovtcov, Y. Ivanov, V. Kim, P. Levchenko, V. Murzin, V. Oreshkin, D. Sosnov, V. Sulimov, L. Uvarov, A. Vorobyev, T. Aushev, K. Ivanov, V. Gavrilov, N. Lychkovskaya, A. Nikitenko, V. Popov, A. Zhokin, M. Chadeeva, R. Chistov, S. Polikarpov, V. Andreev, M. Azarkin, M. Kirakosyan, A. Terkulov, E. Boos, V. Bunichev, M. Dubinin, L. Dudko, A. Gribushin, V. Klyukhin, O. Kodolova, S. Obraztsov, M. Perfilov, V. Savrin, P. Volkov, G. Vorotnikov, V. Blinov, T. Dimova, A. Kozyrev, O. Radchenko, Y. Skovpen, V. Kachanov, D. Konstantinov, S. Slabospitskii, A. Uzunian, A. Babaev, V. Borshch, D. Druzhkin, CMS Collaboration

**Affiliations:** 1 Yerevan Physics Institute, Yerevan, Armenia; 2 Institut für Hochenergiephysik, Vienna, Austria; 3 Universiteit Antwerpen, Antwerp, Belgium; 4 Vrije Universiteit Brussel, Brussels, Belgium; 5 Université Libre de Bruxelles, Brussels, Belgium; 6 Ghent University, Ghent, Belgium; 7 Université Catholique de Louvain, Louvain-la-Neuve, Belgium; 8 Centro Brasileiro de Pesquisas Fisicas, Rio de Janeiro, Brazil; 9 Universidade do Estado do Rio de Janeiro, Rio de Janeiro, Brazil; 10 Universidade Estadual Paulista, Universidade Federal do ABC, São Paulo, Brazil; 11 Institute for Nuclear Research and Nuclear Energy, Bulgarian Academy of Sciences, Sofia, Bulgaria; 12 University of Sofia, Sofia, Bulgaria; 13 Instituto De Alta Investigación, Universidad de Tarapacá, Casilla 7 D, Arica, Chile; 14 Beihang University, Beijing, China; 15 Department of Physics, Tsinghua University, Beijing, China; 16 Institute of High Energy Physics, Beijing, China; 17 State Key Laboratory of Nuclear Physics and Technology, Peking University, Beijing, China; 18 Guangdong Provincial Key Laboratory of Nuclear Science and Guangdong-Hong Kong Joint Laboratory of Quantum Matter, South China Normal University, Guangzhou, China; 19 Sun Yat-Sen University, Guangzhou, China; 20 University of Science and Technology of China, Hefei, China; 21 Nanjing Normal University, Nanjing, China; 22 Institute of Modern Physics and Key Laboratory of Nuclear Physics and Ion-beam Application (MOE), Fudan University, Shanghai, China; 23 Zhejiang University, Hangzhou, Zhejiang China; 24 Universidad de Los Andes, Bogotá, Colombia; 25 Universidad de Antioquia, Medellín, Colombia; 26 Faculty of Electrical Engineering, Mechanical Engineering and Naval Architecture, University of Split, Split, Croatia; 27 Faculty of Science, University of Split, Split, Croatia; 28 Institute Rudjer Boskovic, Zagreb, Croatia; 29 University of Cyprus, Nicosia, Cyprus; 30 Charles University, Prague, Czech Republic; 31 Escuela Politecnica Nacional, Quito, Ecuador; 32 Universidad San Francisco de Quito, Quito, Ecuador; 33 Academy of Scientific Research and Technology of the Arab Republic of Egypt, Egyptian Network of High Energy Physics, Cairo, Egypt; 34 Center for High Energy Physics (CHEP-FU), Fayoum University, El-Fayoum, Egypt; 35 National Institute of Chemical Physics and Biophysics, Tallinn, Estonia; 36 Department of Physics, University of Helsinki, Helsinki, Finland; 37 Helsinki Institute of Physics, Helsinki, Finland; 38 Lappeenranta-Lahti University of Technology, Lappeenranta, Finland; 39 IRFU, CEA, Université Paris-Saclay, Gif-sur-Yvette, France; 40 Laboratoire Leprince-Ringuet, CNRS/IN2P3, Ecole Polytechnique, Institut Polytechnique de Paris, Palaiseau, France; 41 Université de Strasbourg, CNRS, IPHC UMR 7178, Strasbourg, France; 42 Centre de Calcul de l’Institut National de Physique Nucleaire et de Physique des Particules, CNRS/IN2P3, Villeurbanne, France; 43 Institut de Physique des 2 Infinis de Lyon (IP2I), Villeurbanne, France; 44 Georgian Technical University, Tbilisi, Georgia; 45 I. Physikalisches Institut, RWTH Aachen University, Aachen, Germany; 46 III. Physikalisches Institut A, RWTH Aachen University, Aachen, Germany; 47 III. Physikalisches Institut B, RWTH Aachen University, Aachen, Germany; 48 Deutsches Elektronen-Synchrotron, Hamburg, Germany; 49 University of Hamburg, Hamburg, Germany; 50 Karlsruher Institut für Technologie, Karlsruhe, Germany; 51 Institute of Nuclear and Particle Physics (INPP), NCSR Demokritos, Aghia Paraskevi, Greece; 52 National and Kapodistrian University of Athens, Athens, Greece; 53 National Technical University of Athens, Athens, Greece; 54 University of Ioánnina, Ioannina, Greece; 55 HUN-REN Wigner Research Centre for Physics, Budapest, Hungary; 56 MTA-ELTE Lendület CMS Particle and Nuclear Physics Group, Eötvös Loránd University, Budapest, Hungary; 57 Faculty of Informatics, University of Debrecen, Debrecen, Hungary; 58 HUN-REN ATOMKI-Institute of Nuclear Research, Debrecen, Hungary; 59 Karoly Robert Campus, MATE Institute of Technology, Gyongyos, Hungary; 60 Panjab University, Chandigarh, India; 61 University of Delhi, Delhi, India; 62 Saha Institute of Nuclear Physics, HBNI, Kolkata, India; 63 Indian Institute of Technology Madras, Chennai, India; 64 Tata Institute of Fundamental Research-A, Mumbai, India; 65 Tata Institute of Fundamental Research-B, Mumbai, India; 66 National Institute of Science Education and Research, An OCC of Homi Bhabha National Institute, Bhubaneswar, Odisha India; 67 Indian Institute of Science Education and Research (IISER), Pune, India; 68 Isfahan University of Technology, Isfahan, Iran; 69 Institute for Research in Fundamental Sciences (IPM), Tehran, Iran; 70 University College Dublin, Dublin, Ireland; 71 INFN Sezione di Bari, Università di Bari, Politecnico di Bari, Bari, Italy; 72 INFN Sezione di Bologna, Università di Bologna, Bologna, Italy; 73 INFN Sezione di Catania, Università di Catania, Catania, Italy; 74 INFN Sezione di Firenze, Università di Firenze, Florence, Italy; 75 INFN Laboratori Nazionali di Frascati, Frascati, Italy; 76 INFN Sezione di Genova, Università di Genova, Genoa, Italy; 77 INFN Sezione di Milano-Bicocca, Università di Milano-Bicocca, Milan, Italy; 78 INFN Sezione di Napoli, Università di Napoli ‘Federico II’, Naples, Italy; Università della Basilicata, Potenza, Italy, Scuola Superiore Meridionale (SSM), Naples, Italy; 79 INFN Sezione di Padova, Università di Padova, Padua, Italy, Università di Trento, Trento, Italy; 80 INFN Sezione di Pavia, Università di Pavia, Pavia, Italy; 81 INFN Sezione di Perugia, Università di Perugia, Perugia, Italy; 82 INFN Sezione di Pisa, Università di Pisa, Scuola Normale Superiore di Pisa, Pisa, Italy, Università di Siena, Siena, Italy; 83 INFN Sezione di Roma, Sapienza Università di Roma, Rome, Italy; 84 INFN Sezione di Torino, Università di Torino, Turin, Italy, Università del Piemonte Orientale, Novara, Italy; 85 INFN Sezione di Trieste, Università di Trieste, Trieste, Italy; 86 Kyungpook National University, Daegu, Korea; 87 Department of Mathematics and Physics-GWNU, Gangneung, Korea; 88 Institute for Universe and Elementary Particles, Chonnam National University, Kwangju, Korea; 89 Hanyang University, Seoul, Korea; 90 Korea University, Seoul, Korea; 91 Department of Physics, Kyung Hee University, Seoul, Korea; 92 Sejong University, Seoul, Korea; 93 Seoul National University, Seoul, Korea; 94 University of Seoul, Seoul, Korea; 95 Department of Physics, Yonsei University, Seoul, Korea; 96 Sungkyunkwan University, Suwon, Korea; 97 College of Engineering and Technology, American University of the Middle East (AUM), Dasman, Kuwait; 98 Department of Physics, College of Science, Kuwait University, Safat, Kuwait; 99 Riga Technical University, Riga, Latvia; 100 University of Latvia (LU), Riga, Latvia; 101 Vilnius University, Vilnius, Lithuania; 102 National Centre for Particle Physics, Universiti Malaya, Kuala Lumpur, Malaysia; 103 Universidad de Sonora (UNISON), Hermosillo, Mexico; 104 Centro de Investigacion y de Estudios Avanzados del IPN, Mexico City, Mexico; 105 Universidad Iberoamericana, Mexico City, Mexico; 106 Benemerita Universidad Autonoma de Puebla, Puebla, Mexico; 107 University of Montenegro, Podgorica, Montenegro; 108 University of Canterbury, Christchurch, New Zealand; 109 National Centre for Physics, Quaid-I-Azam University, Islamabad, Pakistan; 110 AGH University of Krakow, Kraków, Poland; 111 National Centre for Nuclear Research, Swierk, Poland; 112 Institute of Experimental Physics, Faculty of Physics, University of Warsaw, Warsaw, Poland; 113 Warsaw University of Technology, Warsaw, Poland; 114 Laboratório de Instrumentação e Física Experimental de Partículas, Lisbon, Portugal; 115 Faculty of Physics, University of Belgrade, Belgrade, Serbia; 116 VINCA Institute of Nuclear Sciences, University of Belgrade, Belgrade, Serbia; 117 Centro de Investigaciones Energéticas Medioambientales y Tecnológicas (CIEMAT), Madrid, Spain; 118 Universidad Autónoma de Madrid, Madrid, Spain; 119 Instituto Universitario de Ciencias y Tecnologías Espaciales de Asturias (ICTEA), Universidad de Oviedo, Oviedo, Spain; 120 Instituto de Física de Cantabria (IFCA), CSIC-Universidad de Cantabria, Santander, Spain; 121 University of Colombo, Colombo, Sri Lanka; 122 Department of Physics, University of Ruhuna, Matara, Sri Lanka; 123 CERN, European Organization for Nuclear Research, Geneva, Switzerland; 124 PSI Center for Neutron and Muon Sciences, Villigen, Switzerland; 125 ETH Zurich-Institute for Particle Physics and Astrophysics (IPA), Zurich, Switzerland; 126 Universität Zürich, Zurich, Switzerland; 127 National Central University, Chung-Li, Taiwan; 128 National Taiwan University (NTU), Taipei, Taiwan; 129 High Energy Physics Research Unit, Department of Physics, Faculty of Science, Chulalongkorn University, Bangkok, Thailand; 130 Physics Department, Science and Art Faculty, Çukurova University, Adana, Turkey; 131 Physics Department, Middle East Technical University, Ankara, Turkey; 132 Bogazici University, Istanbul, Turkey; 133 Istanbul Technical University, Istanbul, Turkey; 134 Istanbul University, Istanbul, Turkey; 135 Yildiz Technical University, Istanbul, Turkey; 136 Institute for Scintillation Materials of National Academy of Science of Ukraine, Kharkiv, Ukraine; 137 National Science Centre, Kharkiv Institute of Physics and Technology, Kharkiv, Ukraine; 138 University of Bristol, Bristol, UK; 139 Rutherford Appleton Laboratory, Didcot, UK; 140 Imperial College, London, UK; 141 Brunel University, Uxbridge, UK; 142 Baylor University, Waco, TX USA; 143 Catholic University of America, Washington, DC USA; 144 The University of Alabama, Tuscaloosa, AL USA; 145 Boston University, Boston, MA USA; 146 Brown University, Providence, RI USA; 147 University of California, Davis, Davis, CA USA; 148 University of California, Los Angeles, CA USA; 149 University of California, Riverside, Riverside, CA USA; 150 University of California, San Diego, La Jolla, CA USA; 151 Department of Physics, University of California, Santa Barbara, Santa Barbara, CA USA; 152 California Institute of Technology, Pasadena, CA USA; 153 Carnegie Mellon University, Pittsburgh, PA USA; 154 University of Colorado Boulder, Boulder, CO USA; 155 Cornell University, Ithaca, NY USA; 156 Fermi National Accelerator Laboratory, Batavia, IL USA; 157 University of Florida, Gainesville, FL USA; 158 Florida State University, Tallahassee, FL USA; 159 Florida Institute of Technology, Melbourne, FL USA; 160 University of Illinois Chicago, Chicago, IL USA; 161 The University of Iowa, Iowa City, IA USA; 162 Johns Hopkins University, Baltimore, MD USA; 163 The University of Kansas, Lawrence, KS USA; 164 Kansas State University, Manhattan, KS USA; 165 University of Maryland, College Park, MD USA; 166 Massachusetts Institute of Technology, Cambridge, MA USA; 167 University of Minnesota, Minneapolis, MN USA; 168 University of Nebraska-Lincoln, Lincoln, NE USA; 169 State University of New York at Buffalo, Buffalo, NY USA; 170 Northeastern University, Boston, MA USA; 171 Northwestern University, Evanston, IL USA; 172 University of Notre Dame, Notre Dame, IN USA; 173 The Ohio State University, Columbus, OH USA; 174 Princeton University, Princeton, NJ USA; 175 University of Puerto Rico, Mayagüez, PR USA; 176 Purdue University, West Lafayette, IN USA; 177 Purdue University Northwest, Hammond, IN USA; 178 Rice University, Houston, TX USA; 179 University of Rochester, Rochester, NY USA; 180 Rutgers, The State University of New Jersey, Piscataway, NJ USA; 181 University of Tennessee, Knoxville, TN USA; 182 Texas A&M University, College Station, TX USA; 183 Texas Tech University, Lubbock, TX USA; 184 Vanderbilt University, Nashville, TN USA; 185 University of Virginia, Charlottesville, VA USA; 186 Wayne State University, Detroit, MI USA; 187 University of Wisconsin-Madison, Madison, WI USA; 188 Authors Affiliated with an International Laboratory Covered by a Cooperation Agreement with CERN, Geneva, Switzerland; 189 Authors Affiliated with an Institute Formerly Covered by a Cooperation Agreement with CERN, Geneva, Switzerland; 190 Yerevan State University, Yerevan, Armenia; 191 TU Wien, Vienna, Austria; 192 Ghent University, Ghent, Belgium; 193 Universidade do Estado do Rio de Janeiro, Rio de Janeiro, Brazil; 194 FACAMP-Faculdades de Campinas, São Paulo, Brazil; 195 Universidade Estadual de Campinas, Campinas, Brazil; 196 Federal University of Rio Grande do Sul, Porto Alegre, Brazil; 197 University of Chinese Academy of Sciences, Beijing, China; 198 China Center of Advanced Science and Technology, Beijing, China; 199 University of Chinese Academy of Sciences, Beijing, China; 200 China Spallation Neutron Source, Dongguan, Guangdong China; 201 Henan Normal University, Xinxiang, China; 202 University of Shanghai for Science and Technology, Shanghai, China; 203 The University of Iowa, Iowa City, IA USA; 204 an Institute Formerly Covered by a Cooperation Agreement with CERN, Geneva, Switzerland; 205 Zewail City of Science and Technology, Zewail, Egypt; 206 British University in Egypt, Cairo, Egypt; 207 Ain Shams University, Cairo, Egypt; 208 Purdue University, West Lafayette, IN USA; 209 Université de Haute Alsace, Mulhouse, France; 210 Istinye University, Istanbul, Turkey; 211 an International Laboratory Covered by a Cooperation Agreement with CERN, Geneva, Switzerland; 212 The University of the State of Amazonas, Manaus, Brazil; 213 University of Hamburg, Hamburg, Germany; 214 III. Physikalisches Institut A, RWTH Aachen University, Aachen, Germany; 215 Bergische University Wuppertal (BUW), Wuppertal, Germany; 216 Brandenburg University of Technology, Cottbus, Germany; 217 Forschungszentrum Jülich, Jülich, Germany; 218 CERN, European Organization for Nuclear Research, Geneva, Switzerland; 219 HUN-REN ATOMKI-Institute of Nuclear Research, Debrecen, Hungary; 220 Universitatea Babes-Bolyai-Facultatea de Fizica, Cluj-Napoca, Romania; 221 MTA-ELTE Lendület CMS Particle and Nuclear Physics Group, Eötvös Loránd University, Budapest, Hungary; 222 HUN-REN Wigner Research Centre for Physics, Budapest, Hungary; 223 Physics Department, Faculty of Science, Assiut University, Asyût, Egypt; 224 Punjab Agricultural University, Ludhiana, India; 225 University of Visva-Bharati, Santiniketan, India; 226 Indian Institute of Science (IISc), Bangalore, India; 227 Amity University Uttar Pradesh, Noida, India; 228 IIT Bhubaneswar, Bhubaneswar, India; 229 Institute of Physics, Bhubaneswar, India; 230 University of Hyderabad, Hyderabad, India; 231 Deutsches Elektronen-Synchrotron, Hamburg, Germany; 232 Isfahan University of Technology, Isfahan, Iran; 233 Sharif University of Technology, Tehran, Iran; 234 Department of Physics, University of Science and Technology of Mazandaran, Behshahr, Iran; 235 Department of Physics, Faculty of Science, Arak University, Arak, Iran; 236 Helwan University, Cairo, Egypt; 237 Italian National Agency for New Technologies, Energy and Sustainable Economic Development, Bologna, Italy; 238 Centro Siciliano di Fisica Nucleare e di Struttura Della Materia, Catania, Italy; 239 Università degli Studi Guglielmo Marconi, Rome, Italy; 240 Scuola Superiore Meridionale, Università di Napoli ‘Federico II’, Naples, Italy; 241 Fermi National Accelerator Laboratory, Batavia, IL USA; 242 Consiglio Nazionale delle Ricerche-Istituto Officina dei Materiali, Perugia, Italy; 243 Institut de Physique des 2 Infinis de Lyon (IP2I), Villeurbanne, France; 244 Department of Applied Physics, Faculty of Science and Technology, Universiti Kebangsaan Malaysia, Bangi, Malaysia; 245 Consejo Nacional de Ciencia y Tecnología, Mexico City, Mexico; 246 Trincomalee Campus, Eastern University, Sri Lanka, Nilaveli, Sri Lanka; 247 Saegis Campus, Nugegoda, Sri Lanka; 248 National and Kapodistrian University of Athens, Athens, Greece; 249 Ecole Polytechnique Fédérale Lausanne, Lausanne, Switzerland; 250 University of Vienna, Vienna, Austria; 251 Universität Zürich, Zurich, Switzerland; 252 Stefan Meyer Institute for Subatomic Physics, Vienna, Austria; 253 Laboratoire d’Annecy-le-Vieux de Physique des Particules, IN2P3-CNRS, Annecy-le-Vieux, France; 254 Research Center of Experimental Health Science, Near East University, Mersin, Turkey; 255 Konya Technical University, Konya, Turkey; 256 Izmir Bakircay University, Izmir, Turkey; 257 Adiyaman University, Adiyaman, Turkey; 258 Bozok Universitetesi Rektörlügü, Yozgat, Turkey; 259 Marmara University, Istanbul, Turkey; 260 Milli Savunma University, Istanbul, Turkey; 261 Kafkas University, Kars, Turkey; 262 Istanbul Okan University, Istanbul, Turkey; 263 Hacettepe University, Ankara, Turkey; 264 Erzincan Binali Yildirim University, Erzincan, Turkey; 265 Faculty of Engineering, Istanbul University-Cerrahpasa, Istanbul, Turkey; 266 Yildiz Technical University, Istanbul, Turkey; 267 School of Physics and Astronomy, University of Southampton, Southampton, UK; 268 IPPP Durham University, Durham, UK; 269 Faculty of Science, Monash University, Clayton, Australia; 270 Università di Torino, Turin, Italy; 271 Bethel University, St. Paul, MN USA; 272 Karamanoğlu Mehmetbey University, Karaman, Turkey; 273 California Institute of Technology, Pasadena, CA USA; 274 United States Naval Academy, Annapolis, MD USA; 275 Bingol University, Bingol, Turkey; 276 Georgian Technical University, Tbilisi, Georgia; 277 Sinop University, Sinop, Turkey; 278 Erciyes University, Kayseri, Turkey; 279 Horia Hulubei National Institute of Physics and Nuclear Engineering (IFIN-HH), Bucharest, Romania; 280 Another Institute Formerly Covered by a Cooperation Agreement with CERN, Geneva, Switzerland; 281 Texas A&M University at Qatar, Doha, Qatar; 282 Kyungpook National University, Daegu, Korea; 283 Another International Laboratory Covered by a Cooperation Agreement with CERN, Geneva, Switzerland; 284 Institute of Nuclear Physics of the Uzbekistan Academy of Sciences, Tashkent, Uzbekistan; 285 Another Institute Formerly Covered by a Cooperation Agreement with CERN, Geneva, Switzerland; 286 Northeastern University, Boston, MA USA; 287 Imperial College, London, UK; 288 Yerevan Physics Institute, Yerevan, Armenia; 289 Universiteit Antwerpen, Antwerp, Belgium; 290 CERN, Geneva, Switzerland

## Abstract

Data analyses in particle physics rely on an accurate simulation of particle collisions and a detailed simulation of detector effects to extract physics knowledge from the recorded data. Event generators together with a geant-based simulation of the detectors are used to produce large samples of simulated events for analysis by the LHC experiments. These simulations come at a high computational cost, where the detector simulation and reconstruction algorithms have the largest CPU demands. This article describes how machine-learning (ML) techniques are used to reweight simulated samples obtained with a given set of parameters to samples with different parameters or samples obtained from entirely different simulation programs. The ML reweighting method avoids the need for simulating the detector response multiple times by incorporating the relevant information in a single sample through event weights. Results are presented for reweighting to model variations and higher-order calculations in simulated top quark pair production at the LHC. This ML-based reweighting is an important element of the future computing model of the CMS experiment and will facilitate precision measurements at the High-Luminosity LHC.

## Introduction

In particle physics, Monte Carlo (MC) event generators, paired with a detailed simulation of the experimental apparatus, are ubiquitous. The resulting MC simulations are crucial for simulating the detector response, estimating the acceptance and reconstruction efficiency, and predicting signal and background contributions from different processes expected in data. These are indispensable for obtaining measurements corrected for detector effects that can be directly compared to theoretical predictions. In addition, MC simulations can be used for direct comparisons with collision data to extract parameters of the underlying theory [1].

Large MC samples with billions of events are needed to achieve the physics programme goals of experiments at the CERN LHC, such that the statistical precision of MC samples has a small effect on the total uncertainty of data analyses. The generation and simulation of these MC samples come at a significant computational cost, where the simulation of the detector response and the event reconstruction take more than 75% of the total CPU resources [2, 3]. In addition, the computing resources needed for producing MC samples are expected to increase by factors of ten or more [4] at the High-Luminosity LHC (HL-LHC) [4, 5]. The computing needs of the particle physics programme at the HL-LHC exceed those that can be met by scaling up computing facilities. Current estimates assume that 160 billion fully simulated and reconstructed MC events have to be produced per year after the start of the HL-LHC. This number may become larger by up to 30% because of events with negative weights in next-to-leading order (NLO) and next-to-NLO (NNLO) simulations in quantum chromodynamics (QCD), which reduce the statistical precision of the MC samples. Research and development in several areas are ongoing to meet the strategic goal of minimizing CPU and storage requirements to enable the LHC experiments to make full use of the physics potential offered by the large amount of data expected from the HL-LHC.

An important part of these efforts is a reduction in the storage size and number of MC samples needed for data analyses without compromising the precision and accuracy of analyses. For the evaluation of systematic uncertainties connected to approximations made in the MC event generators, additional samples are typically created. These samples are generated with fewer events than the nominal samples because of limited computing resources. However, the smaller size of these samples can become a limiting factor in precision analyses even with existing data sets. For example, in a recent measurement of the top quark-antiquark ($\text {t}{\bar{\textrm{t}}}$) pair production cross section in proton-proton (pp) collisions at a centre-of-mass energy $\sqrt{s}=13\,\text {Te\hspace{-.08em}V},$ a simultaneous fit of the $\text {t}{\bar{\textrm{t}}}$ cross section and the mass parameter of the top quark in the MC simulation $m_{{\text {t}}}^{\text {MC}}$ is performed [6]. The dominant contribution to the uncertainty in $m_{{\text {t}}}^{\text {MC}}$ arises from the limited statistical precision of the MC samples used for the systematic uncertainty estimation. While a regularization procedure might be considered to smooth out fluctuations, such an approach harbours the risk of weakening genuine physical effects and does not prevent the need to simulate the detector response for additional samples.

In this article, a method is introduced that allows for reweighting MC samples at generator level to represent many relevant aspects from various simulation programs or generated with different parameter values. The reweighting of the entire event is obtained using machine-learning (ML) algorithms, where the output can be stored and applied on a centrally produced MC sample generated with a set of nominal parameter values. In this way, the detailed detector simulation is needed only for one MC sample, which lowers the computational cost. In addition, this approach allows the determination of modelling uncertainties with higher precision because of the increased statistical accuracy of the nominal sample compared to the smaller samples obtained with modelling variations. While the generation of other samples than the nominal one is still needed for the training of the ML model, the detector simulation and reconstruction algorithms do not have to be run on these samples, since the reweighting is done on kinematic variables available at generator level. This saves a considerable amount of CPU resources. In addition, the ML model takes negligible storage space compared to samples with millions of events, such that this approach helps to reduce the data storage needs of particle physics experiments. The user can seamlessly incorporate the trained ML model into the analysis workflow at any stage.

Reweighting can be described in terms of multi-dimensional densities $$p_i.$$ The conditional densities $$p_1(x)$$ and $$p_2(x)$$ describe two densities in a set of given quantities of interest *x*. The density ratio estimate $$p_1(x)/p_2(x) $$ is applied as a weight to the density $$p_2(x)$$ to obtain a conditional density matching $$p_1(x)$$. In standard reweighting approaches, the density ratio estimate is calculated in intervals of *x*,  often realised by binning the two densities in one- or two-dimensional histograms. The density $$p_2(x)$$ is then multiplied by the approximate density ratio estimate in the same intervals of *x* to arrive at a reweighted conditional density which should resemble $$p_1(x)$$. The result is sensitive to the chosen size of the intervals and the dimension of *x* is in practice not larger than two because higher dimensionalities necessitate very large sample sizes. Instead, reweighting techniques based on ML do not have these limitations. The dimension of *x* can be much larger than in standard reweighting methods and the training can be performed by continuously sampling from the initial and target distributions, such that inaccuracies due to binning effects are avoided and correlations between the elements of *x* are accurately reproduced. In addition, ML techniques allow for an interpolation of the reweighted densities in the parameters, resulting in a continuous dependence on the parameters in the simulation of collision events. Several ML methods have been developed in the context of particle physics for reweighting simulations, including boosted decision trees to derive event weights [7], neural networks (NNs) to learn the likelihood ratio between different simulations [8–10], or input convex neural networks [11] to calibrate simulated events [12]. Normalizing flows [13, 14] can be used to learn a mapping between the initial and target distributions, e.g. to calibrate simulation to match data [15], but also to learn the conditional probability distribution of the initial data, from which events following a different conditional distribution can be sampled [16–18]. Reweighting techniques based on ML methods can also enhance the precision of generative adversarial networks [19].

The “deep NN using classification for tuning and reweighting (DCTR)” [9] method can be applied to different scenarios. In this article, the DCTR approach is used for reweighting simulated events of $\text {t}{\bar{\textrm{t}}}$ production to two modelling variations, important for the estimation of systematic uncertainties in the CMS experiment [20]. In addition, the reweighting of an NLO simulation to an NNLO simulation of $\text {t}{\bar{\textrm{t}}}$ production is presented.

The paper is organized as follows. In Sect. 2, a brief description of the ML reweighting method is given. In Sect. 3, the discrete reweighting is presented of a parameter that changes the amount of radiation from the parton shower (PS) in events generated with powheg  v2 [21–23]. The continuous reweighting of a parameter affecting the fragmentation of b quarks in pythia 8 [24] is described in Sect. 4. The predictions obtained from the NLO powheg hvq  [22] event generator are reweighted to the predictions from the powheg MiNNLO  [25–28] event generator at NNLO, both interfaced with pythia 8 for the simulation of the PS and hadronization, in Sect. 5. Finally, the implementation of the method within the CMS analysis and reconstruction software is described in Sect. 6. The paper is summarized in Sect. 7.

## Deep neural network using classification for tuning and reweighting

The DCTR approach is based on a deep NN to reweight MC samples obtained from one simulation to resemble the features of a different simulation or a simulation with varied model parameters. This is achieved by using the full kinematic and flavour information in the event [9]. The method combines an ML architecture for including the particle information [29] with a parameterized classifier [8, 30] to reweight one simulation such that it resembles all relevant features of another. We have chosen this approach for two main reasons. First, it allows for full phase space reweighting, using all the event information such as kinematic and particle type variables and enabling posterior projections onto any desired variable. Second, this method allows for a continuous reweighting as a function of any parameter in the simulation, which is exploited in the b quark fragmentation study in Sect. 4. A full phase space reweighting is important to achieve the best possible precision when estimating modelling uncertainties from a change of parameters in the MC simulation. Its significance becomes particularly pronounced in the context of reweighting from a simulation at NLO to the one at NNLO accuracy. In this scenario, where we have a three-particle phase space (t, $\bar{\textrm{t}}$, and one additional parton) and a four-particle phase space (t, $\bar{\textrm{t}}$, and two additional partons), the method remains effective for all observables derived from the $\text {t}{\bar{\textrm{t}}}$ system using information about the t and $\bar{\textrm{t}}$ quarks, and the combined $\text {t}{\bar{\textrm{t}}}$ system in the training. This stands in contrast to standard reweighting methods, typically involving ratios in bins of two distributions, which might be suboptimal for observables not included in the calculation of these ratios.

### The likelihood ratio trick

The first ingredient of the full phase space reweighting technique is a prescription to derive event weights. Consider two simulations describing the same phase space, denoted as $$\varOmega ,$$ characterized by the probability densities $$p_1(x)$$ and $$p_2(x)$$, where the set of quantities $$x\in \varOmega .$$ Assuming both densities have the same support, the function $$w(x)=p_1(x)/p_2(x) $$ serves as the ideal per-event weight for transforming the second simulation to match the first. This weight function can be effectively approximated by training an ML classifier to discern between the two simulations. For example, an NN function *f*(*x*) is trained using the binary cross-entropy loss

1 $$\begin{aligned} \text {loss}\big (f(x)\big ) = -\sum _{i\in \alpha }\ln f(x_i)-\sum _{i\in \beta }\ln \big (1-f(x_i)\big ), \end{aligned}$$

where $\alpha$ and $\beta$ represent sets of events from the two simulations, i.e. the two classes the binary cross-entropy loss is set up to distinguish between. The likelihood ratio trick states that $f(x)/(1-f(x))\approx p_1(x)/p_2(x)$ [9]. As a consequence, the weight function *w*(*x*),  which is the quantity of interest, can be approximated from the network function *f*(*x*) as $w(x)\approx f(x)/(1-f(x)).$ The benefit of parameterizing *f*(*x*) as an NN is that ML algorithms can take into account the large dimensionality of $\varOmega$ when constructing *f*(*x*). In the case of MC events with weights, for example from simulations at NNLO accuracy, Eq. (1) is modified to become

2 $$\begin{aligned} \text {loss}\big (f(x)\big )&= -\frac{1}{N}\sum _iw^{\text {MC}}_i \Big (t_i\ln f(x_i) \nonumber \\&\quad +(1-t_i)\ln \big (1-f(x_i)\big )\Big ), \end{aligned}$$

where $w^{\text {MC}}_i$ is the MC weight for event *i*,  $t_i$ the true label of an event that is either 1 or 0 depending on whether the event belongs to class 1 or 2, respectively, and $f(x_i)$ is the network function predicting the class of event *i*. A discussion on negative event weights is provided later in Sect. 5.

An important reweighting scenario is when the two simulations have been obtained using the same simulation program but with different parameter values $\theta .$ In this case, $\theta$ becomes the reweighting parameter. For example, when modelling uncertainties are evaluated, one may want to transform $p_{\theta }(x)$ into $p_{\theta +\delta \theta }(x).$ This is equivalent to reweighting an MC sample obtained with the nominal value of a given parameter to match a sample with a variation in this parameter, $\theta +\delta \theta .$ Additionally, the NN reweighting approximation in Eq. (1) can be extended to the continuous case by adding $\theta$ as a parameter to the network function which becomes $f(x,\theta )$ [8, 30]. In this case, the training data are generated with a uniform distribution over a range of $\theta$ and Eq. (1) becomes

3 $$\begin{aligned} \text {loss}\big (f(x,\theta )\big ) = -\sum _{i\in \theta _0}\ln f(x_i,\theta )-\sum _{i\in \varTheta }\ln \big (1-f(x_i,\theta )\big ).\nonumber \\ \end{aligned}$$

Here, $x_i$ is a set of features sampled from a given simulation. The first sum runs over all sets of features in the simulation obtained with the nominal value of the reweighting parameter $\theta _0.$ The second sum runs over all sets of features from different simulations, where the set of these values $\theta$ is denoted by $\varTheta .$ The NN is trained to distinguish between the sample generated with $\theta _0$ and a sample obtained with a set of values $\varTheta .$

### Neural network architecture

The final component of the DCTR method is an NN architecture that can efficiently capture all the crucial aspects of the phase space. We use the particle-flow network (PFN) [29], based on the deep sets framework [31], for this task. The PFN is constructed to include the full event information, such as the particle four-momenta and auxiliary information, like the particle type. The reweighting parameter $\theta$ can also be used to parameterize the NN with the loss function given in Eq. (3). In the work presented here, we only use a small number of particle four-momenta and auxiliary information as input to the PFN, as described below.

The PFN is composed of two interconnected neural networks, *F* and $\varPhi ,$

4 $$\begin{aligned} f(p) = F\left( \sum _{i=1}^N\varPhi (p_i)\right) , \end{aligned}$$

where *p* represents the set of all particles and $p_i$ are the properties of particle *i* (momentum and type), as well as the reweighting parameter $\theta .$ The two networks are integrated into a single model, by sequentially passing data through both networks, with the output layer of $\varPhi$ connected to the input layer of *F*. The network $\varPhi$ processes each particle individually, providing a per-particle internal (latent) representation. The network *F* takes the sum of these latent representations from all particles to produce an overall event-level representation. The dimension of the latent space is determined by the number of dimensions used to represent the input data in a compressed form within the NN. Specifically, the input particles are embedded in an *l*-dimensional latent space using $\varPhi ,$ while *F* is an NN that maps $R^l\rightarrow R.$ The intermediate step involves creating an event-level representation by summing the latent representations of all particles. Finally, the output of the network *F* is a softmax discriminant, which distinguishes between the two classes. More details on the PFN and the setup used in this article are given in Ref. [29].

We set up the NN $\varPhi$ with two hidden layers, each with 100 nodes. The classifier *F* comprises three hidden layers and two output nodes for binary classification, where each hidden layer consists of 100 nodes. The latent space has dimension 128. The ReLU [32] activation function is used for all layers, except for the classification output that uses the softmax function. All models are implemented in keras [33] with the TensorFlow back end [34], passing the data sequentially through both networks. The adam [35] optimization algorithm is used to update the NN parameters during the training for minimizing the cross-entropy loss function for 100 epochs, where each epoch represents a complete pass of the entire training data set through the network. The training and validation loss functions measure the performance of the NN on training and validation data, respectively, checking how well the NN predictions match the true values. To prevent overfitting during training, the “early stopping” method is used to stop the training process if the validation loss does not improve for a certain number of epochs. For example, a patience value of 10 indicates that the training is stopped after 10 epochs without improvement of the validation loss. We use an early stopping with patience values of 10 for both the parton shower matching and b quark fragmentation trainings, while we use patience values of 30 for the MiNNLO training. The learning rate is set to 0.001 in all scenarios. A hyperparameter optimization, specific for each case, is performed for the size of the training inputs and the batch size, where the latter refers to the number of events in the training samples processed simultaneously before the model parameters are updated. To select the best set of hyperparameters, the training is repeated with different choices of hyperparameters and the training giving the best reweighting result is chosen. We find that more complex tasks require a larger batch size, which also depends on the number of events provided as input to the NN. Specific samples have been generated for each of the scenarios discussed in this paper.

## Parton shower matching uncertainties in simulated $\text {t}{\bar{\textrm{t}}}$ production

The CMS experiment uses a setup of powheg +pythia 8 to generate $\text {t}{\bar{\textrm{t}}}$ MC samples that are used in physics analyses. This is done by simulating the hard process of $\text {t}{\bar{\textrm{t}}}$ production with the hvq program [22] in the powheg  v2 [21, 23] generator, which is interfaced with pythia 8.240 [24] for parton showering and hadronization. In this configuration, powheg simulates inclusive $\text {t}{\bar{\textrm{t}}}$ production with matrix elements at NLO accuracy in QCD. A resummation damping factor called $h_{\text {damp}}$ controls the matching of the matrix elements with the PS. It changes the scale of the first emission from the hard process and regulates the radiation at high transverse momentum $p_{\textrm{T}}$. The $h_{\text {damp}}$ parameter enters the simulation in a damping function *D*,  which reduces the real contribution to the Sudakov form factor [36] and is given by

5 $$\begin{aligned} D = \frac{h_{\text {damp}} ^2}{p_{\textrm{T}} ^2+h_{\text {damp}} ^2}. \end{aligned}$$

Here, $p_{\textrm{T}}$ refers to the transverse momentum of the top quark or antiquark and $h_{\text {damp}} =hm_{{\text {t}}},$ where *h* is a real number and $m_{{\text {t}}}$ is the top quark mass of $m_{{\text {t}}} =172.5\,\text {Ge\hspace{-.08em}V}.$ This value is consistent with the most precise result on the top quark mass $m_{{\text {t}}} =172.52\pm 0.33\,\text {Ge\hspace{-.08em}V},$ obtained by the combination [37] of ATLAS and CMS measurements. The parameter $h_{\text {damp}}$ affects the kinematic properties of all particles in the event, most notably the $p_{\textrm{T}}$ of the $\text {t}{\bar{\textrm{t}}}$ system $p_{\textrm{T}} (\text {t}{\bar{\textrm{t}}})$ and its pseudorapidity $\eta (\text {t}{\bar{\textrm{t}}})$, where the definition of the coordinate system is given in Ref. [20].

**Fig. 1:**
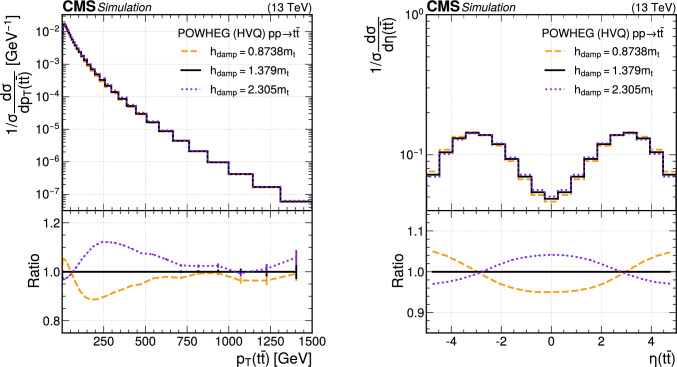
The normalized differential cross section of $\text {t}{\bar{\textrm{t}}}$ production in pp collisions at 13$\,\text {Te\hspace{-.08em}V}$ as a function of the $p_{\textrm{T}}$ (left) and $\eta$ (right) of the $\text {t}{\bar{\textrm{t}}}$ system obtained with the powheg program. The standard setting of $h_{\text {damp}} =1.379\,m_{{\text {t}}}$ (black solid lines) is compared to down (orange dashed lines) and up (violet dotted lines) variations in $h_{\text {damp}}$. The ratios of the predictions with the $h_{\text {damp}}$ variations to the nominal one are shown in the right panels. The vertical bars, in the ratio panels, represent the statistical uncertainties in the MC samples

Since the MC parameter $h_{\text {damp}}$ cannot be derived from first principles, it needs to be determined from measurements. Such estimations result in a sizeable uncertainty in $h_{\text {damp}}$  [38], which can in turn translate into one of the leading systematic uncertainties in precision studies of the top quark. Because the $h_{\text {damp}}$ parameter cannot be reweighted internally by MC generators, additional MC samples obtained for different values of $h_{\text {damp}}$ are necessary to estimate the associated modelling uncertainty. Therefore, the $h_{\text {damp}}$ parameter is an ideal case for a reweighting with the DCTR method.

### The $h_{\text {damp}}$ parameter in CMS

In the CMS experiment, two variations of the $h_{\text {damp}}$ parameter with respect to its nominal value are considered to estimate the uncertainty associated with this parameter. The nominal value of $h_{\text {damp}}$ is set to $1.379\,m_{{\text {t}}}$ [38]. The up and down variations are $2.305\,m_{{\text {t}}}$ and $0.8738\,m_{{\text {t}}},$ obtained from a tune to data. Figure 1 shows the effect of these variations in $h_{\text {damp}}$ for distributions in $p_{\textrm{T}} (\text {t}{\bar{\textrm{t}}})$ and $\eta (\text {t}{\bar{\textrm{t}}})$. Differences in the cross sections up to 10% are observed in the resummation region of $p_{\textrm{T}} (\text {t}{\bar{\textrm{t}}}) \sim 250\,\text {Ge\hspace{-.08em}V}.$ At higher $p_{\textrm{T}} (\text {t}{\bar{\textrm{t}}})$, the effect is negligible and is expected to vanish for $p_{\textrm{T}} (\text {t}{\bar{\textrm{t}}}) >1\,\text {Te\hspace{-.08em}V}$ [39], such that applying an NN weight of 1 is sufficient. A difference of about 5% is observed in $\eta (\text {t}{\bar{\textrm{t}}})$.

In the CMS experiment, the samples generated with the two variations of $h_{\text {damp}}$ are produced with fewer events, less than half the number of events in the nominal sample, where the latter could be of the order of several billion events. The ML reweighting method studied in this paper presents a valuable alternative, ensuring consistent MC statistical uncertainties from both the nominal and variation samples. Since this approach increases the statistical precision of the samples with varied $h_{\text {damp}}$ parameters, it promises to enhance the precision of future analyses.

**Fig. 2:**
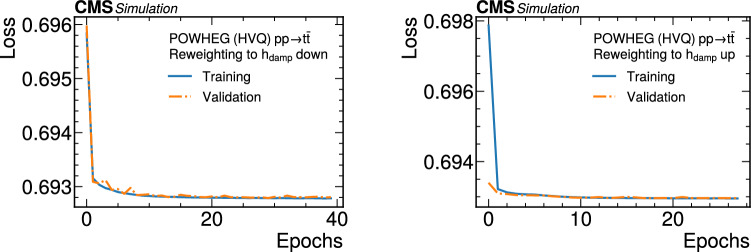
The NN histories of the training for the $h_{\text {damp}}$ parameter reweighting. Shown are the loss functions for the training data (blue solid line) and the validation data (orange dash-dotted line) for the down (left) and up (right) variations of $h_{\text {damp}}$

### Training sample and NN parameters

We train two different NN models to reweight the nominal $h_{\text {damp}}$ MC simulation to the two variations. Each NN model uses a binary classification output to distinguish between the nominal and up/down variations. The NN is trained using the parton-level information at the matrix element level, where the t and $\bar{\textrm{t}}$ information is passed to the NN for training. The particles are represented by their four-momenta and type, where the latter is encoded as a particle ID (PID) number. The set of input variables per particle is given by ($p_{\textrm{T}}$, *y*,  $\phi ,$ *m*,  PID), where *y* denotes the rapidity, $\phi$ the azimuthal angle, and *m* the mass. The inputs to the NN are scaled to values of order $\mathcal {O}(1)$ before the training. The parameter *m* is divided by 244$\,\text {Ge\hspace{-.08em}V}$, which is found to be the maximum value of *m* in the training sample, and $\log (p_{\textrm{T}}/1\,\text {Ge\hspace{-.08em}V})$ is used instead of $p_{\textrm{T}}$. For each setting of the $h_{\text {damp}}$ parameter, 40 million events are generated. Of the resulting 80 million events for the up and down variations in $h_{\text {damp}}$, 75% are used for training and 25% for validation. For both variations in $h_{\text {damp}}$, we find that a batch size of 40,000 events gives the best results.

### Results

The performance of the training is evaluated by examining the values of the loss functions on the training and validation data sets. The graphs of the loss and validation loss functions are shown in Fig. 2, representing the NN models trained with the down (left) and up (right) variations of $h_{\text {damp}}$. The NN has been trained for about 40 epochs for the model trained with the down variation of $h_{\text {damp}}$ and 30 epochs for the model trained with the up variation of $h_{\text {damp}}$.

**Fig. 3:**
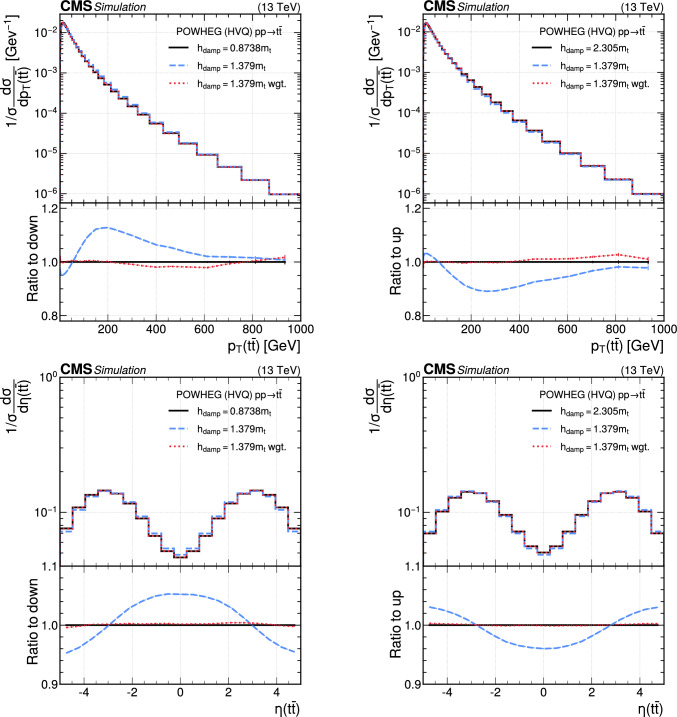
The normalized differential cross section as a function of the $p_{\textrm{T}}$ (upper) and $\eta$ (lower) of the $\text {t}{\bar{\textrm{t}}}$ system. The black solid line shows the predictions from the down (left) and up (right) variations in $h_{\text {damp}}$, and the blue dashed line presents the prediction from the nominal sample. The red dotted line indicates the nominal sample reweighted to the down (left) and up (right) $h_{\text {damp}}$ variations using the DCTR method. The ratios to the samples with the target values of $h_{\text {damp}}$ are displayed in the lower panels, together with their almost negligible statistical uncertainties (vertical error bars)

We test the performance of the reweighting using statistically independent test samples of 100 million events. The weights are found to be close to unity with a standard deviation of about 0.1. No weights smaller than 0.8 and larger than 1.2 are observed. The weights from the trained model are then applied to reweight an MC sample with $h_{\text {damp}} =1.379\,m_{{\text {t}}}$ to the up and down variations with $h_{\text {damp}} =2.305\,m_{{\text {t}}}$ and $0.8739\,m_{{\text {t}}},$ respectively. The effect of the reweighting on the distributions in $p_{\textrm{T}} (\text {t}{\bar{\textrm{t}}})$ and $\eta (\text {t}{\bar{\textrm{t}}})$ is shown in Fig. 3. The accuracy of the reweighting is quantified by the ratio to the nominal sample, shown below each distribution. The target samples, i.e. the samples generated with the variation of $h_{\text {damp}}$, and the reweighted ones agree within 1% in $\eta (\text {t}{\bar{\textrm{t}}})$. Deviations of up to 2% are observed for $p_{\textrm{T}} (\text {t}{\bar{\textrm{t}}}) \gtrsim 400\,\text {Ge\hspace{-.08em}V}.$ Translating this deviation into an uncertainty, the precision of the reweighting is comparable to the statistical precision of a sample with about 250 million events taking into account branching fractions of the $\text {t}{\bar{\textrm{t}}}$ system and typical analysis efficiencies. The effect of the ML reweighting on the $p_{\textrm{T}}$ and *y* distributions of the additional parton has also been checked, reaching the same precision as that of the $\text {t}{\bar{\textrm{t}}}$ system.

**Fig. 4:**
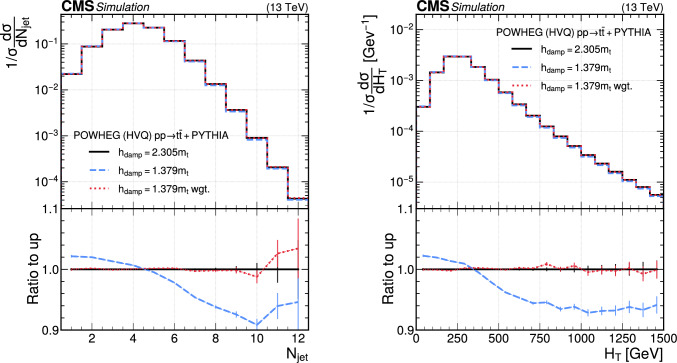
The normalized differential cross section as a function of $N_{\text {jet}}$ (left) and $H_{\textrm{T}}$ (right). The black solid line shows the predictions from the up variation in $h_{\text {damp}}$ and the blue dashed line presents the prediction from the nominal sample. The red dotted line indicates the nominal sample reweighted to the $h_{\text {damp}}$ variation using the DCTR method. The ratios to the target distributions are displayed in the pads below, where the vertical bars represent statistical uncertainties

The accuracy of the reweighting is further tested on $\text {t}{\bar{\textrm{t}}}$ events after the PS and hadronization, where both effects have been simulated with pythia 8. This test is performed to verify that the model trained on parton-level information works for events at the level of stable particles (particle level). Results of the reweighting for distributions obtained at the particle level are shown in Fig. 4. Distributions in the jet multiplicity $N_{\text {jet}}$ and the scalar $p_{\textrm{T}}$ sum of all jets in the event ($H_{\textrm{T}}$) are presented for jets with $p_{\textrm{T}} >30\,\text {Ge\hspace{-.08em}V}$ and $|\eta |<2.4.$ The jets are found using the anti-$k_{\textrm{T}}$ jet algorithm [40] with a distance parameter of $R=0.4.$ The target distributions obtained from samples generated with the up variation of $h_{\text {damp}}$ agree with the reweighted ones within 1% in both observables.

### Statistical uncertainty of the method

**Fig. 5:**
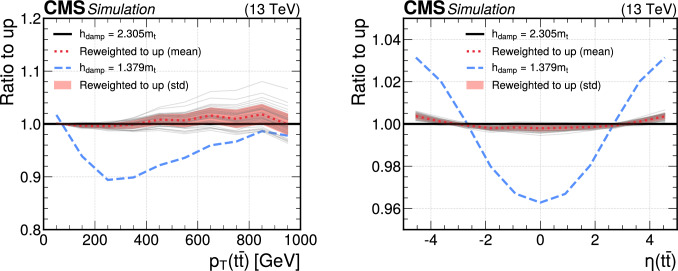
Ratios between the $h_{\text {damp}}$ target distributions in $p_{\textrm{T}} (\text {t}{\bar{\textrm{t}}})$ (left) and $\eta (\text {t}{\bar{\textrm{t}}})$ (right), and 50 different reweightings (grey solid lines). The ratio to the target before the reweighting is shown as a blue dashed line and the mean of the different reweightings as a red dotted line. The red band represents the statistical uncertainty of the method obtained from the standard deviation of the 50 reweighted samples

The statistical precision of the reweighting method is evaluated by repeating the training procedure 50 times [41]. Each training uses 80 million events, randomly chosen from a sample of 200 million events. The results from the 50 different trainings, expressed in ratios of reweighted to target distributions in $p_{\textrm{T}} (\text {t}{\bar{\textrm{t}}})$ and $\eta (\text {t}{\bar{\textrm{t}}})$, are presented in Fig. 5. The average from the 50 reweighted distributions is shown together with the statistical uncertainty, obtained from the standard deviation of these distributions. The statistical uncertainty ranges from less than 0.5% at small values of $p_{\textrm{T}} (\text {t}{\bar{\textrm{t}}})$ up to about 2% at $p_{\textrm{T}} (\text {t}{\bar{\textrm{t}}})$ of about 900$\,\text {Ge\hspace{-.08em}V}$. In $\eta (\text {t}{\bar{\textrm{t}}})$, a statistical uncertainty of less than 1% is found. The target sample, generated with the up variation of $h_{\text {damp}}$, and the average value of the 50 reweighted samples are found to be compatible within the statistical uncertainty of the method, except for $|\eta (\text {t}{\bar{\textrm{t}}}) |>4$ where we observe a difference between the two samples of between one and two standard deviations. This difference is smaller than 0.5%, which is much less than the precision of our simulations and can be neglected. The statistical uncertainty of the method is of the same size as the deviation between the target distribution and the nominal result in Fig. 3.

## The b quark fragmentation

A significant source of uncertainty in precision top quark studies is the fragmentation of b quarks into hadrons [6, 37, 42]. The b quark from the decay ${\text {t}} \rightarrow {\text {W}} {\text {b}}$ fragments into a b hadron and several lighter hadrons. This process is described in pythia by the Lund string model [24], where the probability for the b hadron to carry the momentum fraction *z* of the b quark momentum is given by the Lund–Bowler fragmentation function [43, 44],

6 $$\begin{aligned} f_{{\text {B}}}(z) = \frac{1}{z^{1+br_{{\text {b}}} m_{{\text {b}}} ^2}}(1-z)^a\exp \left( -\frac{bm_{\textrm{T}} ^2}{z}\right) . \end{aligned}$$

The transverse mass of the b hadron is given by $m_{\textrm{T}} ^2=m_{\text {B}} ^2+\Big (p_{\textrm{T}} ^{{\text {B}}} \Big )^2,$ where $m_{\text {B}}$ and $p_{\textrm{T}} ^{{\text {B}}}$ are the b hadron mass and transverse momentum, respectively. The free parameters *a* and *b* are treated as universal for all quarks. Their values $a=0.68$ and $b=0.98\,\text {Ge\hspace{-.08em}V} ^{-2}$ were obtained from a fit to data sensitive to light-quark fragmentation in the Monash tune [45], such as charged-particle multiplicities and momentum fractions. The Lund–Bowler parameter $r_{{\text {b}}}$ and the b quark mass $m_{{\text {b}}}$ are specific to the fragmentation of b quarks, where the latter is set to 4.78$\,\text {Ge\hspace{-.08em}V}$  [45]. In the Monash tune, which is the default for fragmentation in pythia 8, $r_{{\text {b}}} =0.855$ is obtained.

Changing the $r_{{\text {b}}}$ parameter in the event simulation affects certain observables sensitive to the b quark fragmentation. An important example is $x_{{\text {b}}}$, which is defined as the normalized b hadron energy fraction. At the level of stable particles, it is given by [46, 47]

7 $$\begin{aligned} x_{{\text {b}}} = \frac{2p_{{\text {B}}}\cdot p_{{\text {t}}}}{m_{{\text {t}}} ^2}\frac{1}{1-m_{{\text {W}}} ^2/m_{{\text {t}}} ^2}, \end{aligned}$$

where $p_{{\text {B}}}$ and $p_{{\text {t}}}$ are the four-momenta of the b hadron and the top quark, respectively, and $m_{{\text {W}}}$ is the W boson mass. Changes in the value of $r_{{\text {b}}}$ affect the distribution in $x_{{\text {b}}}$, which can lead to efficiency differences in the identification of b jets and can result in sizeable systematic uncertainties in data analyses.

In CMS, the default underlying event tune for 13$\,\text {Te\hspace{-.08em}V}$ analyses is the CP5 tune [38]. Besides other differences from the Monash tune, it uses a smaller value of the strong coupling constant at the mass of the Z boson in the final-state shower, $\alpha _\textrm{S} (M_{{\text {Z}}})=0.118,$ than the Monash tune, which uses $\alpha _\textrm{S} (M_{{\text {Z}}})=0.1365.$ The $r_{{\text {b}}}$ value is not changed in the CP5 tune. Nevertheless, a re-derivation of the $r_{{\text {b}}}$ parameter on top of the CP5 tune with $\textrm{e}^{+}\textrm{e}^{-}$data from LEP results in $r_{{\text {b}}} =1.056\,^{+0.196}_{-0.200}$. In this paper, two NN models are constructed to reweight the sample generated with the CP5 tune with $r_{{\text {b}}} =0.855$ to the nominal and up variation of the $r_{{\text {b}}}$ parameter values of 1.056 and 1.252. For the down variation, the generated value of $r_{{\text {b}}} =0.855$ is used, which almost coincides with $r_{{\text {b}}} =0.856$ obtained from the reanalysis of LEP data. In addition, a continuous reweighting is implemented using ten distinct values of the $r_{{\text {b}}}$ parameter during training. A single NN model is trained to reweight the generated sample to any arbitrary value of $r_{{\text {b}}}$ within the interval [0.6, 1.4],  allowing for an interpolation among the ten $r_{{\text {b}}}$ values.

### Training sample and NN parameters

Approximately one million events for each $r_{{\text {b}}}$ value are used in the discrete reweighting for the training and validation. In the case of the continuous reweighting, the NN is trained on 5 million events, where 2.5 million events were generated with $r_{{\text {b}}} =0.855$ and another 2.5 million events for ten different $r_{{\text {b}}}$ values within the interval [0.6, 1.4],  i.e. 250 000 events were generated for each value of $r_{{\text {b}}}$. In both scenarios, 90% of the events are allocated for training and 10% for validation. The NN architecture is identical to the one described in Sect. 2.2. A batch size of 1000 is found to give optimal results. The NN is trained using the particle-level information of the events produced with pythia 8. In this case, the input variables to the NN are $x_{{\text {b}}}$ of t and $\bar{\textrm{t}}$, as given in Eq. (7), and the value of $r_{{\text {b}}}$ serving as reweighting parameter. While this specific scenario might be manageable with a standard one-dimensional reweighting approach, extending it to a continuous reweighting requires an ML reweighting approach. The observable $x_{{\text {b}}}$ comprises the relevant information from the event, as it includes the four-momenta of the top quarks and the b hadrons in the event. The four-momenta of t and $\bar{\textrm{t}}$ are taken from the last copy in the pythia 8 event record before the decay, and the first copy of the b hadrons are used. The input variable $r_{{\text {b}}}$ is close to unity and the variable $x_{{\text {b}}}$ is defined in the interval [0, 1]. However, while the energy of a b hadron cannot exceed that of the parent bare b quark, this is no longer true in a real collision event, where energy in the hadronization can come from other parts of the final state, such as the underlying event, which is particularly relevant at the LHC. As a result, although most events fall within the range $x_{{\text {b}}} \in [0,1],$ a few values of $x_{{\text {b}}}$ may exceed unity.

### Results

**Fig. 6:**
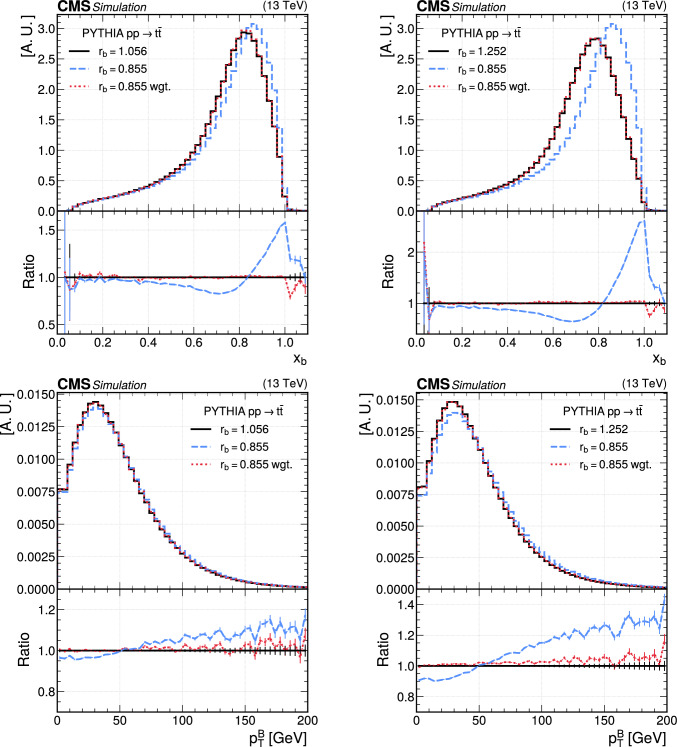
Distributions in $x_{{\text {b}}}$ (upper) and $p_{\textrm{T}} ^{{\text {B}}}$ (lower) from $\text {t}{\bar{\textrm{t}}}$ simulations with pythia 8 with value $r_{{\text {b}}} =0.855$ (dashed blue line) and a second value of $r_{{\text {b}}}$ (solid black line). The nominal sample reweighted to $r_{{\text {b}}} =1.056$ (left) and $r_{{\text {b}}} =1.252$ (right) is shown as red dotted lines. Below each distribution, the ratios to the target distribution are displayed, where the vertical bars represent the statistical uncertainties

The training performance is evaluated by examining the training and validation loss values, where the NN training is stopped after about 20 epochs in both cases. The weights obtained from the reweighting are found to be in the intervals [0.6, 1.4] for the reweighting to $r_{{\text {b}}} =1.056$ and [0.2, 2.5] for the reweighting to $r_{{\text {b}}} =1.252.$ The performance of the reweighting is studied with statistically independent samples with $\sim$2,000,000 events. In particular, the effect of the reweighting on the $x_{{\text {b}}}$ and $p_{\textrm{T}} ^{{\text {B}}}$ distributions is shown in Fig. 6. We find that the reweighted distributions and the distributions simulated with the target $r_{{\text {b}}}$ values agree within the statistical uncertainties.

**Fig. 7:**
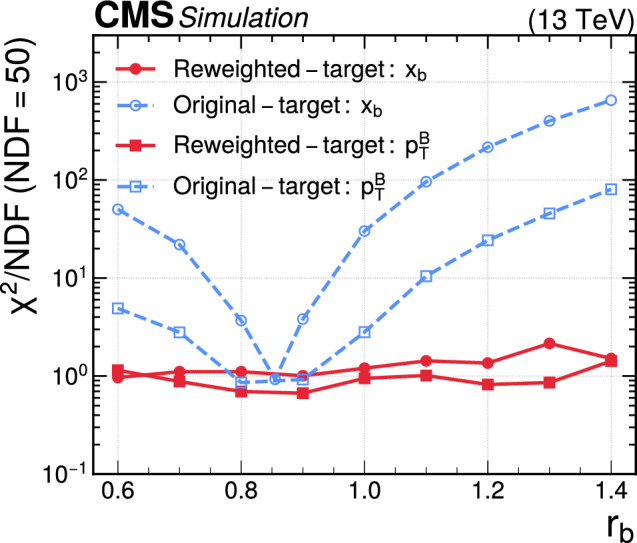
Values of $\chi ^2/\text {NDF}$ obtained for distributions in $x_{{\text {b}}}$ (circles) and $p_{\textrm{T}} ^{{\text {B}}}$ (squares), where target distributions for events with different $r_{{\text {b}}}$ values are compared to a distribution with the nominal value of $r_{{\text {b}}} =0.855$ before the reweighting (blue dashed line) and after the reweighting to the target value of $r_{{\text {b}}}$ (red solid line). The lines connecting the markers are shown for illustration purposes only

The same procedure is applied to the continuous reweighting in $r_{{\text {b}}}$, using statistically independent samples with 250,000 events. To evaluate the goodness of the reweighting for the ten different $r_{{\text {b}}}$ values involved in the training, a $\chi ^2$ test is performed and the results are shown in Fig. 7. This test evaluates the difference between the target distribution generated with a given value of $r_{{\text {b}}}$ and the nominal distribution obtained for $r_{{\text {b}}} =0.855.$ Before the reweighting, $\chi ^2$ values per number of degrees of freedom $(\text {NDF} =50)$ of about 80 for $p_{\textrm{T}} ^{{\text {B}}}$ and 650 for $x_{{\text {b}}}$ are found for the distributions with the largest differences in $r_{{\text {b}}}$ to the nominal value. After the reweighting, we find excellent agreement with the target distributions in $x_{{\text {b}}}$ and $p_{\textrm{T}} ^{{\text {B}}}$ for all points considered in the interval [0.6, 1.4],  with $\chi ^2/\text {NDF}$ values of about unity.

### Statistical uncertainty of the method

**Fig. 8:**
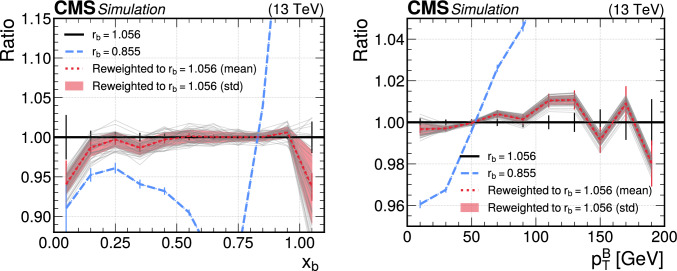
Ratios between the $r_{{\text {b}}}$ target distributions in $x_{{\text {b}}}$ (left) and $p_{\textrm{T}} ^{{\text {B}}}$ (right), and 50 different reweightings (grey solid lines). The ratio to the target before the reweighting is shown as a blue dashed line and the mean of the different reweightings as a red dotted line. The red band represents the statistical uncertainty of the method obtained from the standard deviation of the 50 reweighted samples. The vertical bars show the statistical precision of the samples. In particular, the red bars display the average statistical uncertainty of the 50 reweighted samples

The statistical precision of the discrete reweighting method is evaluated by repeating the training procedure 50 times [41]. Each training uses two million events, randomly chosen from a sample of 26 million events. The results from the 50 different trainings, expressed in ratios of reweighted to target distributions in $x_{{\text {b}}}$ and $p_{\textrm{T}} ^{{\text {B}}}$, are presented in Fig. 8. The average from the 50 reweighted distributions is shown together with the statistical uncertainty, obtained from the standard deviation of these distributions. The statistical uncertainty is a few percent in both distributions. We observe a larger statistical uncertainty of up to about 5% for $x_{{\text {b}}} >1.$ The target sample, generated with $r_{{\text {b}}} =1.056,$ and the average value of the 50 reweighted samples are found to be compatible within the statistical uncertainty of the method, which is of the same order as the deviation between the target distribution and the nominal result in Fig. 6.

## Reweighting to higher-order calculations

Recent theoretical advancements have led to very accurate predictions for $\text {t}{\bar{\textrm{t}}}$ production in pp collisions through fixed-order computations up to NNLO [48–56] accuracy in QCD. These calculations rely on a power expansion in the strong coupling constant and can describe measurements of $\text {t}{\bar{\textrm{t}}}$ production remarkably well [57–71]. However, in specific kinematic regimes, an all-order resummation of radiative corrections is needed for reliable perturbative predictions [72–79]. The PS simulation allows for the inclusion of soft and collinear QCD emissions to all orders in perturbation theory, and resums the large logarithmic corrections. The development of PS algorithms for NLO calculations has resulted in a leap in theoretical accuracy for describing data from the LHC, compared to the previously available leading-order calculations matched to PS simulations. Significant progress has been made recently to combine NNLO calculations with PS MC generators [80–82]. However, $\text {t}{\bar{\textrm{t}}}$ production poses a significant challenge in this respect because of the presence of coloured particles in the initial and final state already at leading order. The MiNNLO_PS_ method [25–28] has been developed specifically for the simulation of $\text {t}{\bar{\textrm{t}}}$ production at NNLO+PS accuracy. This method poses a significant computational complexity and cost for simulating events. The free parameters of the corresponding MC event generator need to be determined, and the results have to be experimentally validated before large-scale simulations can be considered for use in experimental analyses.

In the meantime, corrections for NLO+PS simulations have been derived from a comparison to NNLO calculations. These corrections have been obtained as a function of a single observable, such as the $p_{\textrm{T}}$ of the t or $\bar{\textrm{t}}$ quarks. While these corrections can help to improve the NLO+PS calculations in describing a single distribution [83–85], these corrections fail in capturing the intricate higher-order corrections in the full phase space of $\text {t}{\bar{\textrm{t}}}$ production. We present a reweighting of NLO+PS simulations to NNLO+PS predictions with the DCTR method, which captures the full kinematics of the $\text {t}{\bar{\textrm{t}}}$ system.

### Network training

We perform the reweighting of NLO+PS to NNLO+PS calculations by training a deep NN classifier to distinguish between these two classes of simulations. The event weight for the reweighting is then obtained with the likelihood ratio trick. For this task, 10 million simulated $\text {t}{\bar{\textrm{t}}}$ events at NLO accuracy and 10 million at NNLO accuracy are used, obtained with the hvq  [22] and MiNNLO  [27, 28] package of the powheg  [21, 23] event generator, both interfaced with pythia 8 [24] for the PS. The training data are randomly split into a training set with 75% of the events and a validation set with 25%. Events after the PS are used for the NN training. The input variables to the network are $p_{\textrm{T}}$, *y*,  $\phi ,$ *m* and the particle type of the top quark and antiquark just before the decay, obtained from the last copy in the pythia 8 event record, and the combined $\text {t}{\bar{\textrm{t}}}$ system for each event. All input variables are standardized to take values of the same magnitude. This is achieved by using the expectation values and standard deviations of the corresponding distributions obtained from the NLO simulation. In the case of $p_{\textrm{T}}$, the logarithm of $p_{\textrm{T}}$ is used to reduce the skewness of the input distribution. To this end, we only reweight events based on the kinematics of the $\text {t}{\bar{\textrm{t}}}$ system, inclusive over the additional matrix element and PS radiation.

The divergences from infrared singularities are handled in the powheg method by cancelling soft and collinear real emissions by corresponding virtual corrections. This cancellation leads to a fraction of events with negative weight, which give a negative contribution to physical observables. The fraction of such events with negative weight amounts to about 1% for NLO accuracy and about 10% for NNLO accuracy. However, the binary cross-entropy given by Eq. (2), can become negatively unbounded for negative event weights, making the classification task potentially impossible [86]. In such cases, the loss function can increase without bound, especially when the predicted probabilities are highly confident but incorrect. For example, if the true classification of an event is $t_i=0,$ but the predicted class is given by $f(x_i)=1,$ then Eq. (2) becomes proportional to $-w^{\text {MC}}_i \log (0) = +|w^{\text {MC}}_i |\log (0) \rightarrow -\infty .$ This unbounded growth can lead to extreme values in the loss function, causing numerical instability during the model training process. Consequently, the model may fail to converge to a meaningful solution, rendering the classification task unsolvable. This effect can be mitigated by using a large batch size, which reduces the risk of a single event dominating the loss function. This approach works for NLO simulations, which have a small fraction of negative events. For NNLO calculations with a significant fraction of negative events, the training becomes unstable with the binary cross entropy. A possible solution is the use of the mean square error (MSE) as loss function,

8 $$\begin{aligned} \text {loss}\big (f(x)\big ) = -\frac{1}{N}\sum _i^Nw^{\text {MC}}_i \big (f(x_i)-t_i\big )^2. \end{aligned}$$

In the case of the MSE, negative event weights still result in a reduction of the loss for wrongly classified events. However, the contribution from these events is proportional to $-w^{\text {MC}}_i$ without a large factor, such that the sum over sufficiently many events will result in a total loss function which is positive and not unbounded anymore, stabilizing the training even when including negative events.

A batch size of $2^{17}=131{,}072$ events is found to give the best results for MSE. We reduce the learning rate of 0.001 by 60% when no improvement is achieved after 40% of the training epochs have been processed.

### Results

**Fig. 9:**
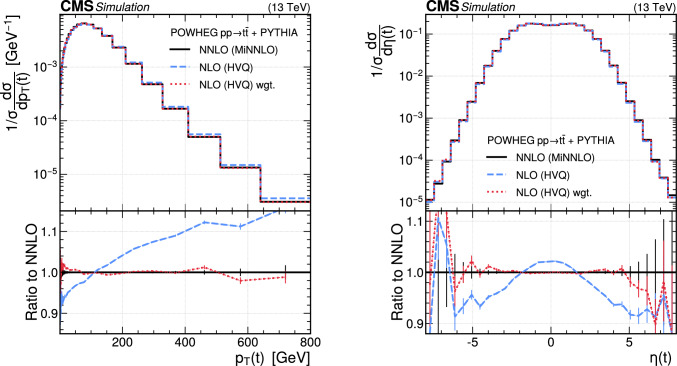
Distributions in top quark $p_{\textrm{T}}$ (left) and $\eta$ (right) obtained from simulations at NNLO accuracy (black solid lines), NLO accuracy (blue dashed lines), and NLO reweighted to NNLO with the DCTR method (red dotted lines). The ratio to the NNLO predictions is shown in the right panels, where the vertical bars correspond to the statistical uncertainties

The results from the NLO-to-NNLO reweighting are presented for about 10 million NLO events and 10 million NNLO events, different from the ones used for the training. Distributions in the kinematic properties of the $\text {t}{\bar{\textrm{t}}}$ system are compared, treating the t and $\bar{\textrm{t}}$ as stable particles. In Fig. 9, the distributions in $p_{\textrm{T}}$ and $\eta$ of the t are shown. The differences between the NLO and NNLO calculations amount to less than 10% at low $p_{\textrm{T}}$, increasing to 15% at high $p_{\textrm{T}}$. The differences between the distributions in $\eta$ are smaller between the NLO and NNLO calculations and range from 2% in the central region to about 6% at $|\eta |=5.$ When comparing the reweighted NLO samples with the NNLO samples, we find agreement within the statistical uncertainties. We observe a very similar picture in the distributions of the $\bar{\textrm{t}}$, which are not shown here.

**Fig. 10:**
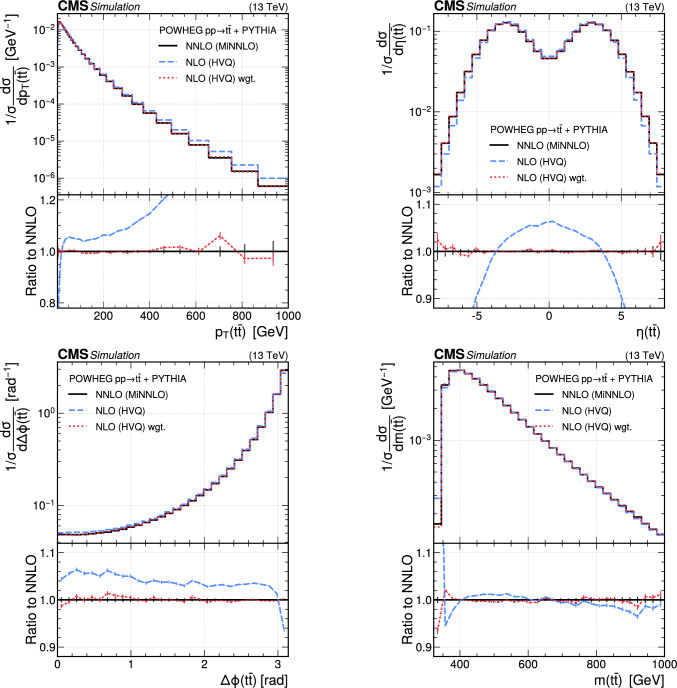
Distributions in $p_{\textrm{T}}$ (upper left), $\eta$ (upper right), $\varDelta \phi$ (lower left), and mass (lower right) of the $\text {t}{\bar{\textrm{t}}}$ system obtained from simulations at NNLO accuracy (black solid lines), NLO accuracy (blue dashed lines), and NLO reweighted to NNLO with the DCTR method (red dotted lines). The ratio to the NNLO predictions is shown in the lower panels, where the vertical bars correspond to the statistical uncertainties

Comparisons between the NLO, NNLO, and NLO-to-NNLO reweighted predictions for the $\text {t}{\bar{\textrm{t}}}$ system are shown in Fig. 10. In all distributions, there are significant differences between the NLO and NNLO predictions. The NLO-to-NNLO reweighting brings the NLO predictions into agreement with the NNLO predictions within the statistical uncertainties over the full kinematic range of the $\text {t}{\bar{\textrm{t}}}$ system. We note that the reweighting works even for observables that were not part of the training, such as $\eta (\text {t}{\bar{\textrm{t}}})$ (upper right of Fig. 10) or $\varDelta \phi ,$ which is the difference in azimuthal angle between the t and $\bar{\textrm{t}}$ (lower left of Fig. 10). The mass of the $\text {t}{\bar{\textrm{t}}}$ system (lower right of Fig. 10) is also in good agreement between the NNLO and NLO-to-NNLO reweighted predictions, which is important in searches for beyond-the-SM effects. The DCTR reweighting achieves this simultaneously with all other kinematic distributions of the $\text {t}{\bar{\textrm{t}}}$ system, which is not possible with traditional reweighting methods. A limitation of the method is that additional radiation beyond the $p_{\textrm{T}}$-leading jet is not accurately reweighted to the NNLO predictions, because the recoil from the $\text {t}{\bar{\textrm{t}}}$ system is distributed evenly over the remaining jets in the event. As a result, observables related to additional radiation such as the jet multiplicity are not accurately reweighted to the NNLO prediction.

**Fig. 11:**
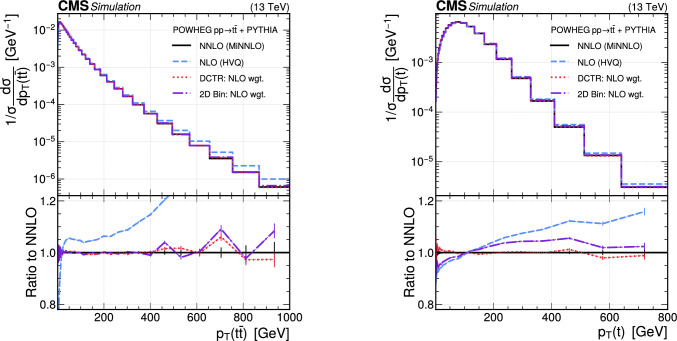
Distributions in $p_{\textrm{T}}$ of the $\text {t}{\bar{\textrm{t}}}$ system (left) and $p_{\textrm{T}}$ of the t (right) obtained from simulations at NNLO accuracy (black solid lines), NLO accuracy (blue dashed lines), NLO reweighted to NNLO with the DCTR method (red dotted lines), and NLO reweighted using a two-dimensional reweighting in $p_{\textrm{T}}$ of the $\text {t}{\bar{\textrm{t}}}$ system and of the t (violet dash-dotted line). The ratio to the NNLO predictions is shown in the right panels, where the vertical bars correspond to the statistical uncertainties

We provide a comparison of the DCTR reweighting with a two-dimensional (2D) reweighting in Fig. 11. The 2D reweighting has been obtained for $p_{\textrm{T}} (\text {t}{\bar{\textrm{t}}})$ and $\eta (\text {t}{\bar{\textrm{t}}})$, where the ratio of NNLO to NLO predictions has been calculated for sufficiently large bins in these two observables to reduce statistical fluctuations. For the distribution in $p_{\textrm{T}} (\text {t}{\bar{\textrm{t}}})$, as shown in Fig. 11 (left), the 2D reweighting works reasonably well over the full range in $p_{\textrm{T}} (\text {t}{\bar{\textrm{t}}})$, where the NLO prediction is brought to agreement with the NNLO prediction within about 2%. However, in the $p_{\textrm{T}}$ of the t, not used in the 2D reweighting and shown in Fig. 11 (right), the 2D reweighting method is not entirely successful. Although the 2D-reweighted simulation brings the NLO prediction closer to the NNLO prediction, there are differences of up to 6%. In contrast, the DCTR reweighting of the NLO sample accurately reproduces the NNLO prediction. The DCTR NLO-to-NNLO reweighting can be used to correct the kinematics of the $\text {t}{\bar{\textrm{t}}}$ system for higher-order effects in the full phase space of $\text {t}{\bar{\textrm{t}}}$ production. The reweighting will have a considerable impact on ongoing and future analyses, until full NNLO+PS simulations such as MiNNLO_PS_ can be produced in very large samples including hadronization effects, particle decays, and detector simulation.

## Implementation in CMSSW

An important aspect of the studies presented in this article is the availability of the trained DCTR models within the central CMS software framework (CMSSW) [87]. The goal of the implementation is to offer all CMSSW users the ability to integrate the trained NN models into physics analyses for the fast evaluation of systematic uncertainties or the impact of higher-order corrections. This encompasses the efficient computation of event weights, specific to each analysis. The computation of these weights can be integrated either at the level of centrally produced data formats or at any chosen stage within the data analysis.

To enhance user flexibility, NN models are stored in the universal open NN exchange (ONNX) format [88]. The ONNX program is an open-source framework, specifically designed to facilitate interoperability and portability between different deep-learning frameworks and tools. Major frameworks like TensorFlow [34], PyTorch [89], and XGBoost [90] support conversion to and from the ONNX format, enabling seamless integration. The runtime of ONNX for inference with trained models is compatible with CMSSW for GPU-based inference. The CMSSW software includes already the required ONNX libraries, eliminating the need for an installation by the user.

In general, the user has to implement the following steps to apply the DCTR reweighting within the CMSSW software.

- Read the nominal sample, i.e. the sample that should be reweighted.
- Load the trained ONNX model.
- Read or calculate the inputs, according to the reweighting scenario.
- Standardize the inputs, identical to what has been done for the training, and pass them to the ONNX model.
- Read out and apply the weights to the nominal sample to obtain the requested variation.

One significant advantage of this workflow lies in the flexibility of weight computation and application throughout various stages of the analysis pipeline. This flexibility enables a reweighting of events at the parton level, as well as the reweighting of events including the detector simulation. Notably, in the latter scenario, the detector simulation has been done only once for the nominal sample which yields a substantial reduction of computational resources, as variations of $h_{\text {damp}}$ and b quark fragmentation parameters merely require reweighting.

## Summary and conclusions

Particle physics relies on the simulation of events using Monte Carlo (MC) event generators for data-to-theory comparisons. Data analyses require the production of several samples simulating the same physical process to estimate systematic uncertainties or the impact of higher-order calculations. To provide statistically significant predictions, these samples have to be very large with billions of events generated and simulated at a high computational cost. Nevertheless, the statistical precision from the finite size of these samples can become a limiting factor in precision analyses. The production of sufficiently large MC samples, such that the statistical precision of these samples is better than the statistical precision of the data, will become increasingly prohibitive at the High-Luminosity LHC (HL-LHC) with the expected computing resources.

In this article, the method “deep neural network using classification for tuning and reweighting (DCTR)” has been introduced to reweight MC samples used in CMS analyses. The weights calculated with the DCTR model enable the modification of one nominal sample to resemble other samples obtained with different parameters or different simulation programs. This methodology avoids the need for simulating the detector response for multiple samples by incorporating the relevant variations in a single sample. While dedicated samples have to be generated for the training and validation of the model, these do not need the full detector simulation and reconstruction, saving up to 75% of the typical CPU resources needed for the production of MC samples in CMS. In addition, after the training of the DCTR model, the training samples can be deleted saving storage space for several billions of events.

The DCTR method has been shown to work reliably for two important sources of modelling uncertainties in the simulation of top quark pair ($\text {t}{\bar{\textrm{t}}}$) production. Currently, the systematic uncertainty connected to the matching of radiation from matrix elements and the parton shower has to be estimated with dedicated samples. The reweighting of variations in the b quark fragmentation shows that a continuous reweighting in a model parameter is possible, paving the way for the determination of model parameters directly from collision data. Additionally, the method has been extended to reweight an NLO simulation to an NNLO one for $\text {t}{\bar{\textrm{t}}}$ production, which will allow for a fast evaluation of the impact of higher-order corrections on data analyses. The DCTR reweighting can be seamlessly integrated into CMS analyses and is already in use by the CMS experiment. A robust performance across a range of scenarios was demonstrated, making the method promising for future applications in other areas as well. For example, it can be extended to other systematic variations or applied to different physics fields beyond top quark studies. It provides an elegant solution to address the computational challenges posed by the production of large MC samples, particularly for the HL-LHC.

## Data Availability

The manuscript has no associated data CERN for the benefit of the CMS Collaboration. [Author’s comment: Release and preservation of data used by the CMS Collaboration as the basis for publications is guided by the CMS data preservation, re-use and open access policy.]

## Code Availability Statement

The manuscript has associated code/software in a data repository. [Author’s comment: The CMS core software is publicly available on GitHub (https://github.com/cms-sw/cmssw) policy (https://doi.org/10.7483/OPENDATA.CMS.1BNU.8V1W)].

## References

[1] A. Buckley et al., Systematic event generator tuning for the LHC. Eur. Phys. J. C **65**, 331 (2010). 10.1140/epjc/s10052-009-1196-7. arXiv:0907.2973

[2] ATLAS Collaboration, ATLAS software and computing HL-LHC roadmap. ATLAS Technical Proposal CERN-LHCC-2022-005 (2022). https://cds.cern.ch/record/2802918

[3] CMS Offline Software and Computing Group, CMS Phase 2 computing model: update document. CMS Note CMS-NOTE-2022-008 (2022). https://cds.cern.ch/record/2815292

[4] HEP Software Foundation, J. Albrecht et al., A roadmap for HEP software and computing R &D for the 2020s. Comput. Softw. Big Sci. **3**, 7 (2019). 10.1007/s41781-018-0018-8. arXiv:1712.06982

[5] G. Apollinari et al., High-luminosity large hadron collider (HL-LHC): technical design report v.0.1. CERN Technical Proposal CERN-2017-007-M (2017). 10.23731/CYRM-2017-004

[6] CMS Collaboration, Measurement of the production cross section, the top quark mass, and the strong coupling constant using dilepton events in pp collisions at TeV. Eur. Phys. J. C **79**, 368 (2019). 10.1140/epjc/s10052-019-6863-8. arXiv:1812.10505 10.1140/epjc/s10052-019-6863-8 PMC6507419 31148943

[7] A. Rogozhnikov, Reweighting with boosted decision trees, in *Proceedings of the 17th International Workshop on Advanced Computing and Analysis Techniques in Physics Research (ACAT 2016): Valparaiso, Chile, January 18–22, 2016* (2016). 10.1088/1742-6596/762/1/012036. arXiv:1608.05806. [J. Phys. Conf. Ser. **762**, 012036 (2016)]

[8] K. Cranmer, J. Pavez, G. Louppe, Approximating likelihood ratios with calibrated discriminative classifiers (2015). arXiv:1506.02169

[9] A. Andreassen, B. Nachman, Neural networks for full phase-space reweighting and parameter tuning. Phys. Rev. D **101**, 091901 (2020). 10.1103/PhysRevD.101.091901. arXiv:1907.08209

[10] B. Nachman, J. Thaler, Neural conditional reweighting. Phys. Rev. D **105**, 076015 (2022). 10.1103/PhysRevD.105.076015. arXiv:2107.08979

[11] B. Amos, L. Xu , J. Zico Kolter, Input convex neural networks, in *Proceedings of the 34th International Conference on Machine Learning (ICML 2017): Sydney, Australia, August 6–11, 2017* (2017). arXiv:1609.07152. https://proceedings.mlr.press/v70/amos17b.html. [PMLR **70**, 146 (2017)]

[12] C. Pollard, P. Windischhofer, Transport away your problems: calibrating stochastic simulations with optimal transport. Nucl. Instrum. Methods A **1027**, 166119 (2022). 10.1016/j.nima.2021.166119. arXiv:2107.08648

[13] E.G. Tabak, C.V. Turner, A family of nonparametric density estimation algorithms. Commun. Pure Appl. Math. **66**, 145 (2013). 10.1002/cpa.21423

[14] E.G. Tabak, E. Vanden-Eijnden, Density estimation by dual ascent of the log-likelihood. Commun. Math. Sci. **8**, 217 (2010). 10.4310/CMS.2010.v8.n1.a11

[15] T. Golling, S. Klein, R. Mastandrea, B. Nachman, Flow-enhanced transportation for anomaly detection. Phys. Rev. D **107**, 096025 (2023). 10.1103/PhysRevD.107.096025. arXiv:2212.11285

[16] J.A. Raine, S. Klein, D. Sengupta, T. Golling, CURTAINs for your sliding window: constructing unobserved regions by transforming adjacent intervals. Front. Big Data **6**, 899345 (2023). 10.3389/fdata.2023.899345. arXiv:2203.09470 37025653 10.3389/fdata.2023.899345 PMC10072325

[17] A. Hallin et al., Classifying anomalies through outer density estimation. Phys. Rev. D **106**, 055006 (2022). 10.1103/PhysRevD.106.055006. arXiv:2109.00546

[18] M. Algren et al., Flow away your differences: conditional normalizing flows as an improvement to reweighting. Submitted to SciPost Phys. (2023). arXiv:2304.14963

[19] S. Diefenbacher et al., DctrGan: improving the precision of generative models with reweighting. JINST **15**, P11004 (2020). 10.1088/1748-0221/15/11/P11004. arXiv:2009.03796

[20] CMS Collaboration, The CMS experiment at the CERN LHC. JINST **3**, S08004 (2008). 10.1088/1748-0221/3/08/S08004

[21] S. Alioli, P. Nason, C. Oleari, E. Re, A general framework for implementing NLO calculations in shower Monte Carlo programs: the powheg box. JHEP **06**, 043 (2010). 10.1007/JHEP06(2010)043. arXiv:1002.2581

[22] S. Frixione, G. Ridolfi, P. Nason, A positive-weight next-to-leading-order Monte Carlo for heavy flavour hadroproduction. JHEP **09**, 126 (2007). 10.1088/1126-6708/2007/09/126. arXiv:0707.3088

[23] S. Frixione, P. Nason, C. Oleari, Matching NLO QCD computations with parton shower simulations: the powheg method. JHEP **11**, 070 (2007). 10.1088/1126-6708/2007/11/070. arXiv:0709.2092

[24] T. Sjöstrand et al., An introduction to PYTHIA 8.2. Comput. Phys. Commun. **191**, 159 (2015). 10.1016/j.cpc.2015.01.024. arXiv:1410.3012

[25] P.F. Monni et al., MiNNLO: a new method to match NNLO QCD to parton showers. JHEP **05**, 143 (2020). 10.1007/JHEP05(2020)143. arXiv:1908.06987. [Erratum: 10.1007/JHEP02(2022)031] 10.1007/JHEP02(2022)031 PMC8827410 35212686

[26] P.F. Monni, E. Re, M. Wiesemann, MiNNLO: optimizing hadronic processes. Eur. Phys. J. C **80**, 1075 (2020). 10.1140/epjc/s10052-020-08658-5. arXiv:2006.04133

[27] J. Mazzitelli et al., Next-to-next-to-leading order event generation for top-quark pair production. Phys. Rev. Lett. **127**, 062001 (2021). 10.1103/PhysRevLett.127.062001. arXiv:2012.14267 34420327 10.1103/PhysRevLett.127.062001

[28] J. Mazzitelli et al., Top-pair production at the LHC with MINNLO. JHEP **04**, 079 (2022). 10.1007/JHEP04(2022)079. arXiv:2112.12135

[29] P. Komiske, E. Metodiev, J. Thaler, Energy flow networks: deep sets for particle jets. JHEP **01**, 121 (2019). 10.1007/JHEP01(2019)121. arXiv:1810.05165

[30] P. Baldi et al., Parameterized neural networks for high-energy physics. Eur. Phys. J. C **76**, 235 (2016). 10.1140/epjc/s10052-016-4099-4. arXiv:1601.07913

[31] M. Zaheer et al., Deep sets, in *Proceedings of the 31st Conference on Neural Information Processing Systems (NIPS 2017): Long Beach CA, USA, December 04–09, 2017* (2017), p. 3391. arXiv:1703.06114. https://papers.nips.cc/paper_files/paper/2017/hash/f22e4747da1aa27e363d86d40ff442fe-Abstract.html

[32] A.F. Agarap, Deep learning using rectified linear units (ReLU) (2018). arXiv:1803.08375

[33] F. Chollet et al., keras (2015). Software available from https://keras.io

[34] M. Abadi et al., TensorFlow: large-scale machine learning on heterogeneous systems (2015). 10.5281/zenodo.4724125. Software available from http://tensorflow.org

[35] D.P. Kingma, J. Ba, adam: a method for stochastic optimization, in *Proceedings of the 3rd International Conference on Learning Representations (ICLR 2015): San Diego CA, USA, May 7–9, 2015* (2015). arXiv:1412.6980

[36] S. Alioli, P. Nason, C. Oleari, E. Re, NLO Higgs boson production via gluon fusion matched with shower in POWHEG. JHEP **04**, 002 (2009). 10.1088/1126-6708/2009/04/002. arXiv:0812.0578

[37] ATLAS and CMS Collaborations, Combination of measurements of the top quark mass from data collected by the ATLAS and CMS experiments at and 8 TeV. Phys. Rev. Lett. **132**, 261902 (2024). 10.1103/PhysRevLett.132.261902. arXiv:2402.08713 10.1103/PhysRevLett.132.261902 38996325

[38] CMS Collaboration, Extraction and validation of a new set of CMS PYTHIA8 tunes from underlying-event measurements. Eur. Phys. J. C **80**, 4 (2020). 10.1140/epjc/s10052-019-7499-4. arXiv:1903.12179 10.1140/epjc/s10052-019-7499-4 PMC6944267 31976986

[39] S. Amoroso et al., Standard model working group report, in *Proceedings of the 11th Les Houches Workshop on Physics at TeV Colliders (PhysTeV 2019): Les Houches, France, June 10–28, 2019* (2020). arXiv:2003.01700

[40] M. Cacciari, G.P. Salam, G. Soyez, The anti-kt jet clustering algorithm. JHEP **04**, 063 (2008). 10.1088/1126-6708/2008/04/063. arXiv:0802.1189

[41] B. Efron, Bootstrap methods: another look at the jackknife, in *Breakthroughs in Statistics*, ed. by S. Kotz, N. Johnson (Springer, New York, 1992), p. 569. 10.1007/978-1-4612-4380-9_41

[42] CMS Collaboration, Measurement of the top quark mass using a profile likelihood approach with the lepton+jets final states in proton–proton collisions at TeV. Eur. Phys. J. C **83**, 963 (2023). 10.1140/epjc/s10052-023-12050-4. arXiv:2302.01967 10.1140/epjc/s10052-023-12050-4 PMC10600315 37906635

[43] C. Bierlich et al., A comprehensive guide to the physics and usage of PYTHIA 8.3. SciPost Phys. Codeb. **8** (2022). 10.21468/SciPostPhysCodeb.8. arXiv:2203.11601

[44] M.G. Bowler, production of heavy quarks in the string model. Z. Phys. C **11**, 169 (1981). 10.1007/BF01574001

[45] P. Skands, S. Carrazza, J. Rojo, Tuning PYTHIA 8.1: the Monash, tune. Eur. Phys. J. C **74**(2014), 3024 (2013). 10.1140/epjc/s10052-014-3024-y. arXiv:1404.5630

[46] G. Corcella, A.D. Mitov, Bottom-quark fragmentation in top-quark decay. Nucl. Phys. B **623**, 247 (2002). 10.1016/S0550-3213(01)00639-3. arXiv:hep-ph/0110319

[47] M. Cacciari, G. Corcella, A.D. Mitov, Soft gluon resummation for bottom fragmentation in top quark decay. JHEP **12**, 015 (2002). 10.1088/1126-6708/2002/12/015. arXiv:hep-ph/0209204

[48] P. Bärnreuther, M. Czakon, A. Mitov, Percent level precision physics at the Tevatron: next-to-next-to-leading order QCD corrections to . Phys. Rev. Lett. **109**, 132001 (2012). 10.1103/PhysRevLett.109.132001. arXiv:1204.5201 23030083 10.1103/PhysRevLett.109.132001

[49] M. Czakon, A. Mitov, NNLO corrections to top-pair production at hadron colliders: the all-fermionic scattering channels. JHEP **12**, 054 (2012). 10.1007/JHEP12(2012)054. arXiv:1207.0236

[50] M. Czakon, A. Mitov, NNLO corrections to top pair production at hadron colliders: the quark-gluon reaction. JHEP **01**, 080 (2013). 10.1007/JHEP01(2013)080. arXiv:1210.6832

[51] M. Czakon, P. Fiedler, A. Mitov, Total top-quark pair-production cross section at hadron colliders through . Phys. Rev. Lett. **110**, 252004 (2013). 10.1103/PhysRevLett.110.252004. arXiv:1303.6254 23829732 10.1103/PhysRevLett.110.252004

[52] M. Czakon, D. Heymes, A. Mitov, High-precision differential predictions for top-quark pairs at the LHC. Phys. Rev. Lett. **116**, 082003 (2016). 10.1103/PhysRevLett.116.082003. arXiv:1511.00549 26967413 10.1103/PhysRevLett.116.082003

[53] M. Czakon, P. Fiedler, D. Heymes, A. Mitov, NNLO QCD predictions for fully-differential top-quark pair production at the Tevatron. JHEP **05**, 034 (2016). 10.1007/JHEP05(2016)034. arXiv:1601.05375

[54] S. Catani et al., Top-quark pair hadroproduction at next-to-next-to-leading order in QCD. Phys. Rev. D **99**, 051501 (2019). 10.1103/PhysRevD.99.051501. arXiv:1901.04005

[55] S. Catani et al., Top-quark pair production at the LHC: fully differential QCD predictions at NNLO. JHEP **07**, 100 (2019). 10.1007/JHEP07(2019)100. arXiv:1906.06535

[56] S. Catani et al., Top-quark pair hadroproduction at NNLO: differential predictions with the mass. JHEP **08**, 027 (2020). 10.1007/JHEP08(2020)027. arXiv:2005.00557

[57] CMS Collaboration, Measurement of differential cross sections for top quark pair production using the lepton+jets final state in proton–proton collisions at 13 TeV. Phys. Rev. D **95**, 092001 (2017). 10.1103/PhysRevD.95.092001. arXiv:1610.04191

[58] CMS Collaboration, Measurement of double-differential cross sections for top quark pair production in pp collisions at TeV and impact on parton distribution functions. Eur. Phys. J. C **77**, 459 (2017). 10.1140/epjc/s10052-017-4984-5. arXiv:1703.01630 10.1140/epjc/s10052-017-4984-5 PMC5587255 28943791

[59] CMS Collaboration, Measurement of normalized differential cross sections in the dilepton channel from pp collisions at TeV. JHEP **04**, 060 (2018). 10.1007/JHEP04(2018)060. arXiv:1708.07638

[60] CMS Collaboration, Measurements of differential cross sections of top quark pair production as a function of kinematic event variables in proton–proton collisions at TeV. JHEP **06**, 002 (2018). 10.1007/JHEP06(2018)002. arXiv:1803.03991

[61] CMS Collaboration, Measurements of differential cross sections in proton–proton collisions at TeV using events containing two leptons. JHEP **02**, 149 (2019). 10.1007/JHEP02(2019)149. arXiv:1811.06625

[62] CMS Collaboration, Measurement of the top quark mass in the all-jets final state at TeV and combination with the lepton+jets channel. Eur. Phys. J. C **79**, 313 (2019). 10.1140/epjc/s10052-019-6788-2. arXiv:1812.10534 10.1140/epjc/s10052-019-6788-2 PMC6454813 31031568

[63] CMS Collaboration, Measurement of normalised multi-differential cross sections in pp collisions at , and simultaneous determination of the strong coupling strength, top quark pole mass, and parton distribution functions. Eur. Phys. J. C **80**, 658 (2020). 10.1140/epjc/s10052-020-7917-7. arXiv:1904.05237

[64] CMS Collaboration, Measurement of differential production cross sections in the full kinematic range using lepton+jets events from proton–proton collisions at TeV. Phys. Rev. D **104**, 092013 (2021). 10.1103/PhysRevD.104.092013. arXiv:2108.02803

[65] ATLAS Collaboration, Measurements of top-quark pair differential cross-sections in the e channel in pp collisions at TeV using the ATLAS detector. Eur. Phys. J. C **76**, 538 (2016). 10.1140/epjc/s10052-016-4366-4. arXiv:1511.04716 10.1140/epjc/s10052-016-4366-4 PMC5501225 28747847

[66] ATLAS Collaboration, Measurement of top quark pair differential cross-sections in the dilepton channel in pp collisions at and 8 TeV with ATLAS. Phys. Rev. D **94**, 092003 (2016). 10.1103/PhysRevD.94.092003. arXiv:1607.07281. [Addendum: 10.1103/PhysRevD.101.119901]

[67] ATLAS Collaboration, Measurements of top-quark pair differential cross-sections in the lepton + jets channel in pp collisions at TeV using the ATLAS detector. Eur. Phys. J. C **77**, 292 (2017). 10.1140/epjc/s10052-017-4821-x. arXiv:1612.05220

[68] ATLAS Collaboration, Measurements of top-quark pair differential cross-sections in the lepton+jets channel in pp collisions at TeV using the ATLAS detector. JHEP **11**, 191 (2017). 10.1007/JHEP11(2017)191. arXiv:1708.00727

[69] ATLAS Collaboration, Measurement of lepton differential distributions and the top quark mass in production in pp collisions at TeV with the ATLAS detector. Eur. Phys. J. C **77**, 804 (2017). 10.1140/epjc/s10052-017-5349-9. arXiv:1709.09407 10.1140/epjc/s10052-017-5349-9 PMC6956925 31999283

[70] ATLAS Collaboration, Measurements of top-quark pair differential and double-differential cross-sections in the channel with pp collisions at TeV using the ATLAS detector. Eur. Phys. J. C **79**, 1028 (2019). 10.1140/epjc/s10052-019-7525-6. arXiv:1908.07305. [Erratum: 10.1140/epjc/s10052-020-08541-3]

[71] ATLAS Collaboration, Measurement of the production cross-section and lepton differential distributions in dilepton events from pp collisions at TeV with the ATLAS detector. Eur. Phys. J. C **80**, 528 (2020). 10.1140/epjc/s10052-020-7907-9. arXiv:1910.08819

[72] M. Beneke, P. Falgari, S. Klein, C. Schwinn, Hadronic top-quark pair production with NNLL threshold resummation. Nucl. Phys. B **855**, 695 (2012). 10.1016/j.nuclphysb.2011.10.021. arXiv:1109.1536

[73] M. Beneke et al., Inclusive top-pair production phenomenology with TOPIXS. JHEP **07**, 194 (2012). 10.1007/JHEP07(2012)194. arXiv:1206.2454

[74] H.X. Zhu et al., Transverse-momentum resummation for top-quark pairs at hadron colliders. Phys. Rev. Lett. **110**, 082001 (2013). 10.1103/PhysRevLett.110.082001. arXiv:1208.5774 23473132 10.1103/PhysRevLett.110.082001

[75] H.T. Li et al., Top quark pair production at small transverse momentum in hadronic collisions. Phys. Rev. D **88**, 074004 (2013). 10.1103/PhysRevD.88.074004. arXiv:1307.2464

[76] S. Catani, M. Grazzini, A. Torre, Transverse-momentum resummation for heavy-quark hadroproduction. Nucl. Phys. B **890**, 518 (2014). 10.1016/j.nuclphysb.2014.11.019. arXiv:1408.4564

[77] S. Catani, M. Grazzini, H. Sargsyan, Transverse-momentum resummation for top-quark pair production at the LHC. JHEP **11**, 061 (2018). 10.1007/JHEP11(2018)061. arXiv:1806.01601

[78] W.-L. Ju et al., Top quark pair production near threshold: single/double distributions and mass determination. JHEP **06**, 158 (2020). 10.1007/JHEP06(2020)158. arXiv:2004.03088

[79] S. Alioli, A. Broggio, M.A. Lim, Zero-jettiness resummation for top-quark pair production at the LHC. JHEP **01**, 066 (2022). 10.1007/JHEP01(2022)066. arXiv:2111.03632

[80] K. Hamilton, P. Nason, C. Oleari, G. Zanderighi, Merging H/W/Z+0 and 1 jet at NLO with no merging scale: a path to parton shower + NNLO matching. JHEP **05**, 082 (2013). 10.1007/JHEP05(2013)082. arXiv:1212.4504

[81] S. Alioli et al., Matching fully differential NNLO calculations and parton showers. JHEP **06**, 089 (2014). 10.1007/JHEP06(2014)089. arXiv:1311.0286

[82] S. Höche, Y. Li, S. Prestel, Drell–Yan lepton pair production at NNLO QCD with parton showers. Phys. Rev. D **91**, 074015 (2015). 10.1103/PhysRevD.91.074015. arXiv:1405.3607

[83] CMS Collaboration, Search for a heavy resonance decaying to a top quark and a W boson at TeV in the fully hadronic final state. JHEP **12**, 106 (2021). 10.1007/JHEP12(2021)106. arXiv:2104.12853

[84] CMS Collaboration, Search for a heavy resonance decaying into a top quark and a W boson in the lepton+jets final state at TeV. JHEP **04**, 048 (2022). 10.1007/JHEP04(2022)048. arXiv:2111.10216

[85] ATLAS Collaboration, Measurements of differential cross-sections in top-quark pair events with a high transverse momentum top quark and limits on beyond the standard model contributions to top-quark pair production with the ATLAS detector at TeV. JHEP **06**, 063 (2022). 10.1007/JHEP06(2022)063. arXiv:2202.12134

[86] B. Nachman, J. Thaler, Neural resampler for Monte Carlo reweighting with preserved uncertainties. Phys. Rev. D **102**, 076004 (2020). 10.1103/PhysRevD.102.076004. arXiv:2007.11586

[87] CMS core software repository (CMSSW). 10.7483/OPENDATA.CMS.1BNU.8V1W

[88] Open neural network exchange (ONNX). Software available at https://onnx.ai

[89] A. Paszke et al., PyTorch: an imperative style, high-performance deep learning library, in *Proceedings of the 33rd Conference on Neural Information Processing Systems (NeurIPS 2019): Vancouver, Canada, December 08–14, 2019* (2019). arXiv:1912.01703

[90] T. Chen, C. Guestrin, XGBoost: a scalable tree boosting system, in *Proceedings of the 22nd ACM SIGKDD International Conference on Knowledge Discovery and Data Mining: San Francisco CA, USA, August 13–17, 2016* (2016). 10.1145/2939672.2939785. arXiv:1603.02754

